# Generation of High Yielding and Fragrant Rice (*Oryza sativa* L.) Lines by CRISPR/Cas9 Targeted Mutagenesis of Three Homoeologs of Cytochrome P450 Gene Family and *OsBADH2* and Transcriptome and Proteome Profiling of Revealed Changes Triggered by Mutations

**DOI:** 10.3390/plants9060788

**Published:** 2020-06-23

**Authors:** Babar Usman, Gul Nawaz, Neng Zhao, Yaoguang Liu, Rongbai Li

**Affiliations:** 1 College of Agriculture, State Key Laboratory for Conservation and Utilization of Subtropical Agro-Bioresources, Guangxi University, Nanning 530004, China; babarusman119@gmail.com (B.U.); gulnawazmalik@yahoo.com (G.N.); nengzhao_gxu@163.com (N.Z.); 2 State Key Laboratory for Conservation and Utilization of Subtropical Agricultural Bioresources, South China Agricultural University, Guangzhou 510642, China

**Keywords:** rice, yield, cytochrome P450, CRISPR/Cas9, mutations, transcriptome, proteome

## Abstract

The significant increase in grain yield and quality are often antagonistic but a constant demand for breeders and consumers. Some genes related to cytochrome P450 family are known for rice organ growth but their role in controlling grain yield is still unknown. Here, we generated new rice mutants with high yield and improved aroma by simultaneously editing three cytochrome P450 homoeologs (*Os03g0603100*, *Os03g0568400*, and *GL3.2*) and *OsBADH2* with the CRISPR/Cas9 system, and RNA-sequencing and proteomic analysis were performed to unveil the subsequent changes. High mutation efficiency was achieved in both target sites of each gene and the mutations were predominantly only deletions, while insertions were rare, and no mutations were detected in the five most likely off-target sites against each sgRNA. Mutants exhibited increased grain size, 2-acetyl-1-pyrroline (2AP) content, and grain cell numbers while there was no change in other agronomic traits. Transgene-DNA-free mutant lines appeared with a frequency of 44.44% and homozygous mutations were stably transmitted, and bi-allelic and heterozygous mutations followed Mendelian inheritance, while the inheritance of chimeric mutations was unpredictable. Deep RNA sequencing and proteomic results revealed the regulation of genes and proteins related to cytochrome P450 family, grain size and development, and cell cycle. The KEGG and hub-gene and protein network analysis showed that the gene and proteins related to ribosomal and photosynthesis pathways were mainly enriched, respectively. Our findings provide a broad and detailed basis to understand the role of CRISPR/Cas9 in rice yield and quality improvement.

## 1. Introduction

Rice is considered one of the most common staple food crops which feeds more than half of the world’s population. However, its sustainable production is facing challenges, including resource competition, such as water and land, climate changes, farm labor shortages, and the rising cost of production. An efficient breeding system to boost the crop yield is required to overcome the food demand of speedily expanding the human population [1]. Especially, fragrant rice is popular worldwide and has higher market rates because of the fragrance of its grains. Therefore, to improve the rice yield and quality together, it is vital to adopt innovative breeding approaches. Seed size is one of the most significant agronomic characters in crop domestication and seeds are considered as the most important food resources for human beings. Understanding and evaluating the role of different gene families will be effective to promote rice breeding to produce high yielding and environment-friendly varieties. Rice grain weight is defined by a combination of grain width, length, and thickness. Many genes, such as *DEP1* [2], *Gn1a* [3], *GS3* [4], *GW2* [5], *qSW5* [6], *GW5* [7], *GS5* [8], *GW8* [9], *GL7*/*GW7* [10], *Ghd7* [11], *GIF1* [12], and *PROG1* [13], have been found to be regulating the grain size by stimulation of cell cycle machinery in promoting cell division. The combination of cell division and expansion determines the growth of plant organs [14]. Grain size is determined by the development of the embryo, endosperm, and maternal tissues, but the molecular mechanism underlying seed growth is largely unclear in many plant species. To explore the new genes responsible for the regulation of cellular growth and division and promoting grain size is worthwhile in rice breeding. Previous studies proved that the desirable mutations in yield-associated loci under natural conditions are favorable [3,4,5,6,15,16,17,18].

Hundreds of volatile compounds have been found in the flavor of boiled aromatic rice, among them, 2-acetyl-1-pyrroline (2AP) is mainly responsible for fragrant phenotype [19]. The detailed biosynthesis pathway of the 2AP compound has not yet been completely elucidated. In recent years, some studies have reported genetic analysis and utilization of aroma genes and found that a single recessive gene *fgr* (*OsBADH2*), which encodes betaine aldehyde dehydrogenase 2 (BADH2), is responsible for fragrance in rice. Sequence alignment of the *fgr* gene between non-fragrant and fragrant rice varieties identified mutations in exon regions [20,21]. Mutations in *OsBADH2* resulted in a fragrant phenotype by enhancing 2AP biosynthesis [22].

Cytochrome P450s (Cyt P450s) represent the biggest family in plants that play an important role in different biochemical pathways, plant metabolism, cell proliferation, and expansion [23,24]. Previous studies have estimated that there are 356 Cyt P450 genes and 99 associated pseudogenes in the rice [23]. In Arabidopsis, several positive regulators of seed size have been characterized, including *KLU*/*CYTOCHROME P450 78A5* (*CYP78A5*) and its homolog *CYP78A7* acts through a non-cell-autonomous signal to promotes organ growth [25,26]. Functional analysis showed that *CYP78A9* acts redundantly with *EOD3*/*CYP78A6* and *CYP78A8* and controls seed size and outer integument growth [27,28]. *PLASTOCHRON1* (*PLA1*) belongs to *CYP78A* member in rice which controls the leaf initiation timing and the termination of vegetative growth by encoding *CYP78A11* [29]. In rice, *BG2,* and its paralogue *GL3.2* encodes Cyt P450, *OsCYP78A13* are responsible for grain size and variations in the exon regions of these genes determined the difference in grain yield [30]. These studies suggest that the Cyt P450 family is involved in controlling plant organ growth and holds great potential in rice breeding to increase grain yield; however, their downstream mobile signal has not yet been identified.

To investigate the gene functions, several loss-of-function and gain-of-function mutants have been generated in rice [31,32]. According to the previous studies, approximately 30% of rice genes belong to redundant gene families with unknown functions [33]. In recent years, advanced mutagenesis approaches are largely used to screen the phenotype of interest such as yield improvement. Conventional hybrid breeding is laborious, expensive, time-consuming, and takes several generations followed by continuous crossing and backcrossing to transfer the natural mutant gene into cultivated varieties. Transgenic breeding using modern mutagenesis approaches is an alternative approach to generate candidate lines with inheritable homozygous mutations over generations. The clustered regularly interspaced short palindromic repeats (CRISPR)-Cas9 (CRISPR-associated protein 9) genome editing technology has successfully applied in many plant species to quickly address the emerging challenges in agriculture [34,35]. It can be used to precisely edit plant genome sequences to achieve the desired traits and has significant advantages compared to other genome editing technologies [34,36]. Application of CRISPR-Cas9 technology in editing the plant genome for improving plant protection, abiotic, and biotic stress tolerance and yield has made remarkable progress [15,16,17,18,34,35]. The CRISPR-Cas9-mediated mutations in *Gn1a*, *DEP1*, *GS3*, *IPA1* [37], *GW2* [38], and *GW5* [39] resulted in increased yield of rice plants, revealing the potential of the CRISPR-Cas9 system for creating novel allelic variations for developing high yielding crops.

To perceive the effects of mutations on plant genome, high-throughput profiling of transcripts or proteins is an effective technique to explore the diverse changes in complex biological processes [40]. With the dramatic advancement in molecular biology techniques, large-scale transcriptome profiling and iTRAQ (isobaric tags for relative and absolute quantification)-based proteome analysis may provide a way to find a global view of gene and protein expression patterns and provides insights into the potential molecular mechanism of mutagenesis generating [41,42,43]. In rice, joint analysis of multiple omics data has been widely used to understand the gene networks involved in plant growth in response to hormones, induced callus differentiation of mature seeds, and metabolism of mitochondrial arginine under anaerobic environment [44,45,46]. Integrative omics analysis has been largely used in many plant species, but no transcriptome and proteome data exist to understand the molecular mechanism underlying the enhanced grain yield in CRISPR/Cas9 generated rice mutants.

In the present work, several novel rice mutants were generated by editing three homoeologs of the Cyt P450 family (*Os03g0603100*, *Os03g0568400*, and *GL3.2*) and *OsBADH2* via CRISPR/Cas9 system, and comparative transcriptome profiling and proteomic analysis was performed to study the revealed changes caused by mutations. Our study found that the mutant plants exhibited enlarged grain size, increased grain cell numbers, and showed fragrant phenotype. The results showed that mutations in each gene resulted in increased grain weight while triple mutants in which three Cyt P450 genes were edited simultaneously, exhibited higher yield compared to the single gene mutants and wild type (WT). Transcriptome and proteomic analysis found that the genes and proteins related to cell division, Cyt P450, and grain size were differentially expressed in mutant plants. Our findings suggest that Cyt P450 genes may play a role in controlling grain size by stimulating cell cycle machinery in rice. In summary, our work suggests that the CRISPR/Cas9-based mutagenesis in Cyt P450 genes hold great potential in rice breeding to improve the rice grain yield.

## 2. Results

### 2.1. Plasmid Construction and Analysis of Mutant Plants

#### 2.1.1. Construction of CRISPR-Cas9 Expression Vector

**Figure 1:**
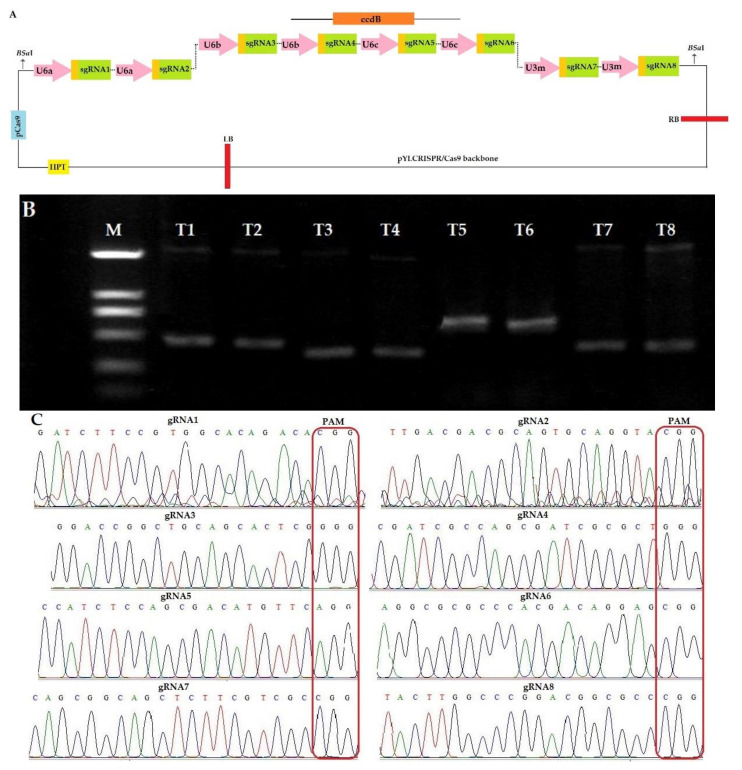
Illustration of target sites and assembly of single guided RNAs (sgRNAs) in pYLCRISPR/Cas9PubiH. (**A**) The illustration of eight sgRNAs in pYLCRISPR/Cas9. (**B**) Product length of all sgRNAs in the second round of PCR, M; molecular marker (2000 bp), T1-T8 represents target1-target8 (**C**) Sequencing confirmation of eight sgRNAs in pYLCRISPR/Cas9PubiH, PAM, protospacer adjacent motif.

The sequencing results of target genes in IR-96 variety showed that *Os03g0603100* and *Os03g0568400* showed nucleotide polymorphism after aligning with the reference genome (Figure S1). The sgRNAs for these two genes were selected according to the sequencing results. The generation of sgRNA expression cassettes by ligation of the target adaptors to the BsaI-digested sgRNA intermediate plasmids followed by PCR and amplified fragments were assembled with the Golden Gate ligation method. The expression cassette was linked by using the Pps-GGL and Pgs-GGR primers (Table S1). The first sgRNA was linked to the B-L site and eighth sgRNA sequence to the B-R site which was used to amplify the ligated sgRNA expression cassette. The sgRNA1 and sgRNA2 were followed by U6a, sgRNA3 and sgRNA4 followed by U6b, sgRNA5 and sgRNA6 followed by U6c, sgRNA7 and sgRNA8 followed by U3m promoter sites (Figure 1A). Expression cassette was generated by overlapping PCR and the amplified product was purified by using TaKaRa MiniBEST Purification Kit Ver.4.0. The size of the amplified product for sgRNA1, sgRNA2, sgRNA3, sgRNA4, sgRNA5, sgRNA6, sgRNA7, and sgRNA8 was 629 bp, 629 bp, 515 bp, 515 bp, 767 bp, 767 bp, 564 bp and 564 bp, respectively (Figure 1B). The eight sgRNA sequences were ligated to plant expression vector pYLCRISPR/Cas9Pubi-H to form a recombinant vector. The successfully constructed recombinant vector was transformed into Agrobacterium EHA105 by heat shock and then the bacterial liquid was amplified by PCR with the detection primers (SP-L1/SP-R) (Table S1). The bacterial solution amplified to the target fragment size was directly sequenced and all sgRNA sequences were confirmed (Figure 1C). The CRISPR/Cas9 binary vector was constructed and a transformation experiment was performed.

#### 2.1.2. Identification of Mutant Plants and Sequencing Analysis of Mutation Sites

**Figure 2:**
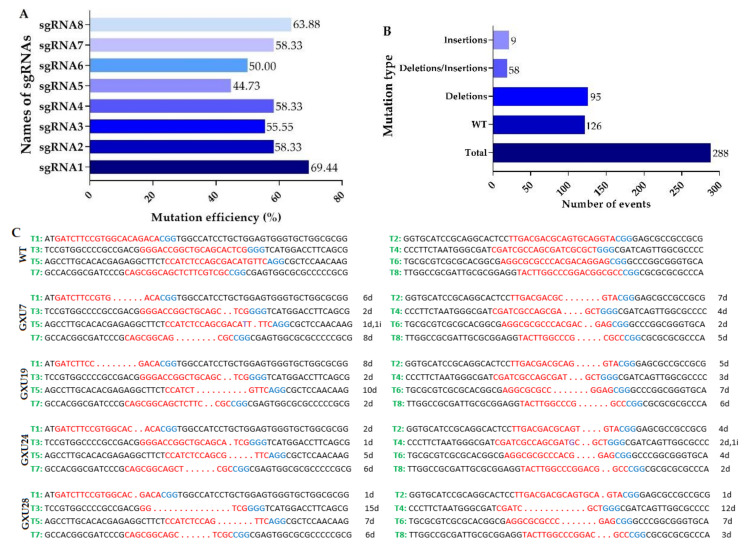
The CRISPR/Cas9 system induced mutation frequency detection in T_0_ generation and sequence alignment of heritable homozygous rice mutants. (**A**) The mutation efficiency of each sgRNA; (**B**) types of mutations with the number of mutation events; (**C**) PCR identification and sequence alignment of wild-type (WT) and four homozygous T_0_ mutant lines (i.e., GXU7, GXU19, GXU24, and GXU28). T1–T8 represents Target1-Target8. Red letters are the sgRNA sites; letter in violet color represents inserted nucleotide sequence; the blue letters are protospacer adjacent motif (PAM) sequence; “d” and “i” indicates the deletions and insertion, respectively.

We treated 150 calli with transformed *A. tumefaciens* and obtained a total of 60 rice plantlets. The PCR reaction was performed using target specific primers, and a total of 36 mutant plants were screened showing mutations at target sites. We analyzed the mutant plants by direct sequencing of PCR products containing the target sites and mutations types were identified using Degenerate Sequence Decoding (DSDecode). The plantlets were considered as mutants which showed mutations at least one target site. The mutation efficiency of target1 (T1), target2 (T2), target3 (T3), target4 (T4), target5 (T5), target6 (T6), target7 (T7), and target8 (T8) was 69.44%, 58.33%, 55.55%, 58.33%, 44.73%, 50.0%, 58.33% and 63.88%, respectively (Figure 2A). Mono-allelic heterozygous mutations resulted frequently, while bi-allelic heterozygous and chimeric mutations were rare (Table 1). Most of the mutations were only deletions, while only insertions were very rare (Figure 2B; Table S2). We also tracked the segregation of T-DNA in the T_1_ population of the T_0_ plants. In T_1_ generation, total, 16 T-DNA-free plants were obtained, of which four mutant plants (GXU7, GXU19, GXU24, and GXU28) were homozygous for all target sites. This confirmed that T_1_ rice plants could be utilized to effectively develop T-DNA-free rice plants, as the pYLCRISPR/Cas9 vector and the mutations are inherited independently of one another. We selected these homozygous mutant plants for further investigations. GXU7 showed homozygous mutations with −6 bp, −7 bp, −2 bp, −4 bp, −1 bp and +1 bp, −2 bp, −8 bp and −5 bp deletions and insertions on T1, T2, T3, T4, T5, T6, T7, and T8 target sites, respectively. Homozygous mutant GXU19 showed −8 bp, −5 bp, −2 bp, −3 bp, −10 bp, −7 bp, −2 bp, and −6 bp deletions on T1, T2, T3, T4, T5, T6, T7, and T8 target sites, respectively. The mutant line GXU24 resulted in homozygous mutations with −2 bp, −4 bp, −1 bp, −2 bp and +1 bp, −5 bp, −4 bp, −6 bp and -2 bp deletions on T1, T2, T3, T4, T5, T6, T7, and T8 target sites, respectively (Figure 2C). GXU28 showed homozygous mutation with −1 bp, −1 bp, −15 bp, −12 bp, −7 bp, −7 bp, −6 bp and −3 bp deletion on T1, T2, T3, T4, T5, T6, T7, and T8 target sites, respectively. To investigate the pattern of transmission of CRISPR/Cas9-mediated targeted gene modification in homozygous mutant plants, we obtained T_1_, T_2_, and T_3_ generations by strict self-pollination and used them for testing of targeted mutations. For each line, three plants were randomly selected and examined by using target-specific primers. The results revealed that, T-DNA-free homozygous mutants (i.e., GXU7, GXU19, GXU24, and GXU28) showed constant and heritable mutations in T_1_ and, subsequently, in the T_2_ and T_3_ generations (Figure S2). Five putative off-targets were examined by PCR-based sanger sequencing in T_0_ generation, and the results revealed that there was no off-target effect was found for all targets (Table S3).

#### 2.1.3. Agronomic Performance Assessment of Wild-Type and Mutant Lines

**Figure 3:**
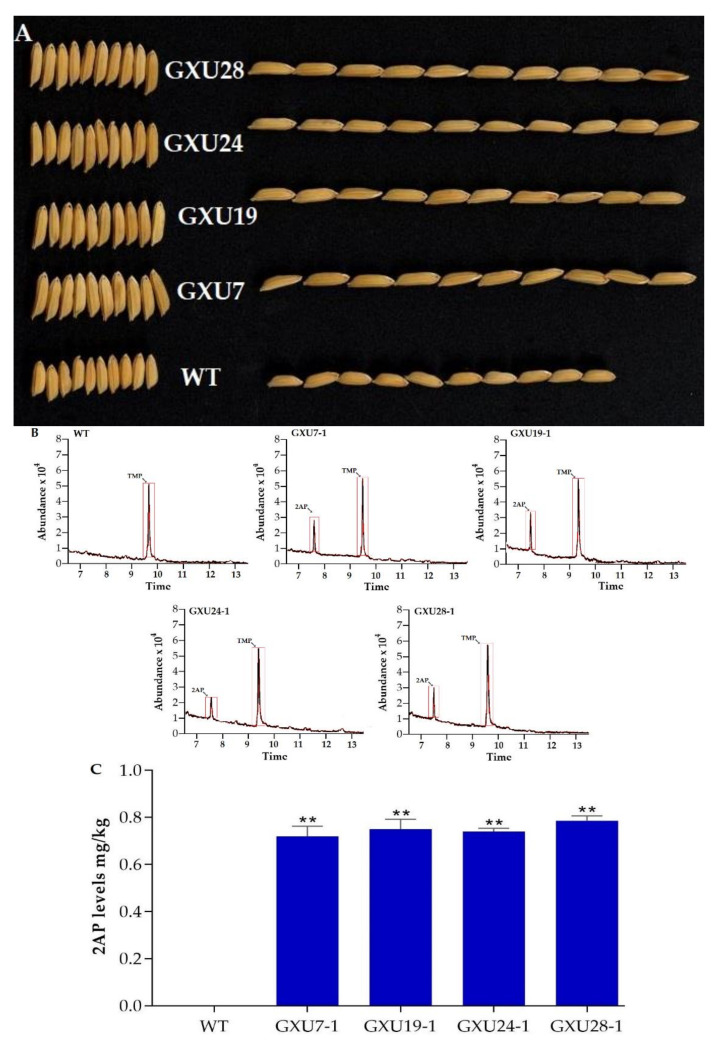
Grain phenotype and the 2-acetyl-1-pyrroline (2AP) levels of homozygous mutants and wild-type (WT) grains. (**A**) Grain phenotype of WT and homozygous mutant lines (GXU7-1, GXU19-1, GXU24-1, and GXU27-1). (**B**) The total ion chromatograms (TIC) of 2AP and TMP (as internal standard) in the T_1_ homozygous mutant lines and WT. (**C**) 2AP levels of the mutants and WT. Values are means ± SD, *n* = 5, *p ≤* 0.01, student’s *t*-test. ** indicates significant difference.

We recorded 1000-grain weight (GWT) in T_0_ generation and that the mutations in single target site result in a slight or non-significant increase in GWT while the mutations in both targets sites and three genes of Cyt P450 gene family resulted in increased GWT in mutant lines as compared to WT (Table S4). Non-Significant difference was found in mutant lines GXU4, GXU6, GXU8, GXU25, and GXU27 with GWT of 29.1 g, 28.6 g, 29.1 g, 28.8 g, and 28.9 g, respectively. GXU4 showed only homozygous mutation at first and heterozygous mutation at the sixth target site, whereas, GXU8 showed heterozygous mutations at the first, fourth, and eight target sites. GXU6, GXU25, and GXU27 showed heterozygous mutations at the first, fourth and third target sites, respectively. A significant difference was obtained for mutant lines GXU7, GXU19, GXU24, and GXU28 with homozygous mutations at all target sites with GWT of 34.5 g, 33.9 g, 34.6 g, and 34.9 g, respectively, while WT showed 28.0 g of GWT in T_0_ generation. These results revealed a strong association between mutation frequency and GWT. We selected homozygous mutant lines GXU7, GXU19, GXU24 and GXU28 for recording agronomic data and the results of T_1_, T_2_, and T_3_ generation showed that the grain width (GWD) and grain length (GL) of mutant plants significantly increased from 3.4 mm to 4.4 mm and 9.2 mm to 11.5 mm, respectively (Table 2; Figure 3A). As expected, the GWT of mutant lines was also increased from 28.0 g to 34.9 g. However, WT and mutant lines showed no significant difference in the other main agronomic traits including the plant height (PH), grain number per plant (GNPP), and seed setting rate (SSR) (Table 2).

#### 2.1.4. Determination of 2-AP Content in Wild-Type and Homozygous Mutant Lines

The biosynthesis of 2-AP determined the fragrance level of rice grain. The rice seeds of homozygous mutant lines from the T_1_ generation were assessed for 2AP content by GC-MS. Results showed that the 2AP peak was detected from all the homozygous lines (GXU7-1, GXU19-1, GXU24-1, and GXU28-1) but WT showed no peak (Figure 3B). The 2AP levels in the mutant lines were significantly produced. Homozygous lines GXU7-1, GXU19-1, GXU24-1, and GXU28-1 showed 0.72 mg/kg, 0.75 mg/kg, 0.74 mg/kg, and 0.78 mg/kg 2AP levels, respectively (Figure 3C).

#### 2.1.5. Segregation Ratio of Mutant Plants

Transmission of the targeted mutations induced by CRISPR/Cas9 were investigated by self-fertilization of T_0_ mutants and subjected to segregation analyses. The T_1_ progeny of homozygous plants (GXU7-1) exhibited homozygosity for all target sites with the same mutations. These results indicate that homozygous mutations were stably transmitted from T_0_ to T_1_ generation for all target sites. We observed the segregation pattern of a heterozygous mutation in GXU1-1 for first, second, fourth, fifth, seventh, and eight target sites and in GXU2-1 for third and sixth target sites. The T_1_ progeny (GXU1-1 and GXU2-1) of T_0_ heterozygous lines was segregated according to Mendelian inheritance and resulted in homozygous and heterozygous mutations and WT plants. The progeny of the bi-allelic mutant line GXU30-1 followed the classic Mendelian inheritance (1:2:1). The segregation of chimeric mutations was detected in the GXU35-1 line. The results showed that the inheritance pattern of chimeric mutations was less predictable and more diverse (Table S5).

#### 2.1.6. Anatomical Observation of WT and Mutant Line (GXU7-1) Grain

**Figure 4:**
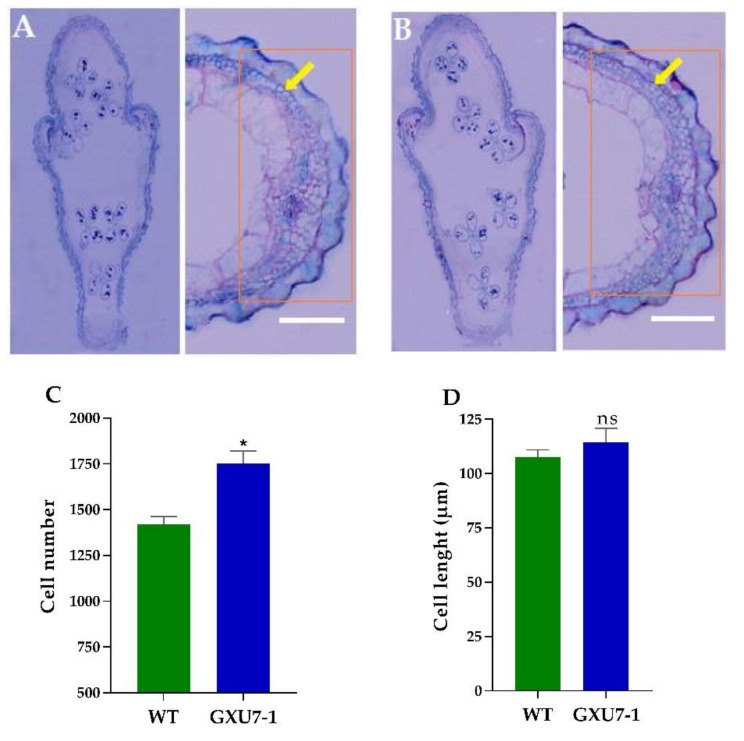
Anatomical observation and comparison of wild-type (WT) and mutant line (GXU7-1) grain cells. Transverse section of whole grain and paraffin section of the developed lemmas of WT (**A**) and GXU7-1 (**B**), respectively. (**C**) Cell number of the lemma. (**D**) Cell length of the outer epidermal lemma. Data are given as means ± SD, *n* = 5, *p* ≤ 0.01. * and ns represents significant and non-significant difference, respectively.

Rice grain size is usually determined by the size and number of glume cells. To clarify the reason, why the mutant plants showed large grain size, we observed the paraffin sections of the WT and the mutant line (GXU7-1) at the heading stage and before flowering (Figure 4A). It was found that there was no significant change in the size of the cells in the glume shell between the mutant line (GXU7-1) and WT, but the number of cells in GXU7-1 were increased significantly. The number of cells increased were from 1420 in WT to 1750 in GXU7-1, an increase of 23.24% (Figure 4B). The cell length of WT and GXU7-1 was 107 µm and 114 µm, respectively (Figure 4C). From this, we concluded that the larger grain size of the GXU7-1 mutant was caused by an increase in the number of cells.

### 2.2. Transcriptome Analysis

#### 2.2.1. Outcome of RNA-Sequencing Analysis

The RNA-seq was performed to gather information about the transcriptional events involved in the response to CRISPR/Cas9 mutations. We sequenced the two RNA-Seq groups (WT and GXU7-1) with three replicates, which almost generated 10,661,415 and 10,597,095 raw sequencing reads, for WT and GXU7-1, respectively. After filtering low-quality reads, we obtained 10,511,127 and 10,503,372 clean reads for WT and GXU7-1, respectively (Table S6). Total transcripts of 26,999 genes were detected in pre-filtration and 22,204 genes were obtained after filter cutoff of 100 with normal log2 (*n* + pseudo-count). The box-and-whisker plot of transcriptome count data distribution showed no difference among medians of each replicate but a clear difference between WT and mutant line (GXU7-1) (Figure S3A). The histogram about count data distribution showed that the frequency of proteins was higher at 4 to 12 of log_2_ (counts + 1). The correlation coefficient for WT and GXU7-1 was 1 and 0.92, respectively (Figure S3B). After performing the analysis with an adjusted *p*-value cutoff of 0.05 and a minimum fold change of 1.2, a total of 2122 DEGs (413 upregulated and 1709 downregulated) were identified between WT and GXU7-1 (Figure S3C).

We selected twenty most variable genes with highest mean expression values and found that eight genes including *OsAKR2* (Aldo–keto reductase), *rbcL* (RuBisCO large subunit), *Os03g0227700* (Cyt P450 90B2), *Os08g0512600* (B-type plant-specific cyclin-dependent kinase), *Os05g0135700* (S-adenosylmethionine synthetase 1), *Os01g0791033* (Similar to ribulose-1,5-bisphosphate carboxylase/oxygenase large subunit), *Os09g0346500* (Similar to Chlorophyll a-b binding protein, chloroplast precursor; LHCP) and *Os12g0274700* (small subunit of Rubisco) showed higher expression level in mutant line than WT. Seven genes including *Os12g0291100* (Petunia ribulose 1,5-bisphosphate carboxylase small subunit mRNA), *Os11g0171300* (Fructose-bisphosphate aldolase; ALDP), *Os02g0626100* (Similar to Phenylalanine ammonia-lyase), psbB (Photosystem II CP47 reaction center protein), *Os08g0200300* (Photosystem II), *Os01g0600900* (chlorophyll a-b binding protein 2, chloroplast precursor; LHCP) and *Os02g0216300* (Similar to cDNA) showed lower expression level in mutant line than WT, while five genes including *psbA* (Photosystem II protein D1), *cob* (Cyt b), *Os12g0291400* (Similar to Petunia ribulose 1,5 bisphosphate carboxylase small subunit mRNA), *Os02g0626400* (Phenylalanine ammonia-lyase) and *Os01g0501800* (Similar to Photosystem II oxygen-evolving complex protein 1) showed a similar pattern of gene expression in WT and mutant line (Figure S3D).

We also manually examined the whole list of DEGs to find the genes related to Cyt P450 family, grain size, and cyclin. We found that seven genes related to Cyt P450 were downregulated and only one gene was upregulated in GXU7-1. Intriguingly, we found that genes including *GIF2* (*grain incomplete filling 2*), *Os02g0762600* (*SG1-SHORT GRAIN1-related protein*), *Os09g0517600* (regulation of grain size) and *OspPLAIIIα* (*patatin-related phospholipase A*) were significantly downregulated in GXU7-1. Moreover, we found that four rice cyclin genes including *CycB2;1, CYCD4, CycD4;2* and *CycP4;1* were significantly upregulated while *CycB1;4* gene was downregulated in GXU7-1. We found twenty-five DEGs related to cyclin-like F-box domain-containing protein, among them twenty-two were downregulated and only three were upregulated in GXU7-1. There were three DEGs related to cyclin-dependent protein kinase, among them two were upregulated and one was downregulated in GXU7-1 (Table S7).

#### 2.2.2. Functional Classification and Pathway Enrichment of DEGs.

Gene ontology (GO) enrichment analysis was carried out to further illustrate the main biological functions of DEGs. All the DEGs can be divided into three categories, including molecular function (MF), biological process (BP), and cellular component (CC). The significantly enriched categories were presented at the user threshold of *p*  ≤ 0.05. GO annotation of all 2122 DEGs related to CC category were mainly related to the membrane-enclosed lumen, organelle lumen, intracellular organelle lumen, and nuclear part. The BP category was classified as cellular nitrogen compound metabolic process, nucleic acid metabolic process, cellular aromatic compound metabolic process and heterocycle metabolic process. MF Regarding MF, the DEGs were only related to double-stranded DNA binding and a total of 291 genes were involved in this category (Figure S4A).

Pathway enrichment analysis was performed to estimate the number of DEGs contained at a different level and significantly enriched pathways were presented. Kyoto Encyclopedia of Genes and Genomes (KEGG) KEGG annotation revealed that the DEGs were enriched in ribosomal pathways (Figure S4A). The KEGG pathway graph rendered by Pathview showed that the DEGs involved in ribosomal pathways were upregulated in mutant plants (Figure S4B).

#### 2.2.3. Hub-Gene Network Analysis

The data was imported to the Cytoscape plugin CytoHubba application and the top 10 genes were evaluated. Our analyses revealed that putative ribosomal protein L13a (*Os03g0755700*), putative acidic ribosomal protein P2a-2 (*Os01g0191100*), 60S ribosomal protein L32 (Os09g0501200), putative 60S ribosomal protein L44 (*Os07g0523100*), 40S ribosomal protein S19 (*Os03g0424500*), putative 60S acidic ribosomal protein P2A (*Os02g0529700*), 40S ribosomal protein S26 (*Os05g0477300*), putative tRNA-glutamine synthetase (*Os01g0185200*), 40S ribosomal protein S30-like (*Os02g0804100*) and 60S ribosomal protein L29 (*Os05g0355500*) were the top ten hub-genes among all DEPs (Figure S5).

### 2.3. Proteomic Analysis

#### 2.3.1. Basic Information and Analysis of Identified Peptides and Proteins

We identified the 40,859 peptides from 543,998 spectra, corresponding to 6597 proteins in the samples of WT and GXU7-1. After filtering the missing count values and 100 filter cutoff, we obtained 5979 and 5956 proteins, respectively. The box and whisker plot of count data showed a clear difference between WT and GXU7-1 (Figure S6A). The histogram of proteins count data showed that the protein frequency was higher at 6 to 11 of log_2_ (counts + 1) (Figure S6B). After performing the analysis with an adjusted *p*-value of 0.05 and a minimum fold change of 1.2, a total of 104 DEPs (84 upregulated and 20 downregulated) were obtained (Figure S6C). The information about all identified proteins is given in Additional file.

The top twenty proteins selected on the basis of mean expression value revealed that fourteen proteins including A0A0P0Y838 (similar to ribulose-1,5-bisphosphate carboxylase/oxygenase large subunit), A0A088BVM4 (ribulose bisphosphate carboxylase large chain), Q0J7P4 (putative HAP3 subunit of the CCAAT box-binding transcription factor), Q10HD0 (similar to photosystem II type II chlorophyll a/b binding protein), A0A0K2JL42 (protein containing a placenta-specific 8 domain), Q6YUX0 (transcription factor ILI5), Q9SNK3 (similar to glyceraldehyde-3-phosphate dehydrogenase), Q943W1 (similar to photosystem II oxygen-evolving complex protein 1), B9F4Q9 (E3 ubiquitin-protein ligase GW2), Q69RJ0 (ferredoxin-dependent glutamate synthase), Q40677 (fructose-bisphosphate aldolase), E9KIM9 (photosystem II CP43 reaction center protein) and A0A0P0WP33 (phosphoglycerate kinase) and Q6ZIK5 (growth-regulating factor 4) showed higher expression level in mutant line than WT, while six proteins including P93431 (ribulose bisphosphate carboxylase/oxygenase activase), P18566 (petunia ribulose 1,5-bisphosphate carboxylase small subunit mRNA), E9KIM8 (Photosystem II D2 protein), Q0D5P8 (similar to oxygen-evolving enhancer protein 3-2), A0A0K2JL38 (cadmium resistance protein) and Q7 × 8A1 (glyceraldehyde-3-phosphate dehydrogenase) showed similar expression patterns (Figure S6D).

We also manually searched the DEPs that were related to the Cyt P450 family, rice grain development, and cell cycle. We found DEPs (Q94IW5 and Q6F4F5) related to the Cyt P450 family, among them, Q94IW5 was upregulated and Q6F4F5 was downregulated in GXU7-1. As expected, the DEPs related to grain size and development were also identified, among them Q6YUX0 (positive regulator of grain length 2, Q6ZIK5 (grain size on chromosome 2), Q6YYV8 (BAHD acyltransferase-like protein gene) and B9F4Q9 (E3 ubiquitin-protein ligase GW2) were upregulated while Q6AT90 (transcription factor APG) was downregulated in GXU7-1. Three DEPs including Q0J4I1 (cyclin-dependent kinase B2-1), Q6YXH8 (cyclin-D4-1), and P29618 (cyclin-dependent kinase A-1) controlling cell cycle and division were found to be significantly upregulated in GXU7-1 (Table S8).

#### 2.3.2. Gene Ontology (GO) and Pathway Enrichment Analysis of DEPs

DEPs were categorized according to BP, CC and MF to gain insight into the functional categories. GO annotations for DEPs related to BP were mainly associated with plant-type cell wall biogenesis, carbohydrate metabolic process and plant-type cell wall organization or biogenesis. Proteins conferring CC were mainly associated with photosystem I, plastoglobule, photosystem and organelle subcompartment. Finally, from the MF perspective, the DEPs were mainly involved in chlorophyll-binding, structural constituent of cytoskeleton, cellulose synthase (UDP-forming) activity and cellulose synthase activity (Figure S7A).

KEGG analysis revealed that the DEPs were only enriched in photosynthesis pathways (Figure S7A). The DEPs (Lhca1, Lhca2, and Lhcb2) related to photosynthesis- antenna proteins were upregulated in mutant plants (Figure S7B).

#### 2.3.3. Hub-Protein Analysis

Top 10 hub-proteins were identified with a high degree of connectivity in the protein-protein interaction (PPI) network. The analysis for top-ten hub proteins revealed that the putative photosystem II core complex proteins psbY (Q6ZJ41), plastocyanin, chloroplastic (Q0DFC9), photosystem I reaction center subunit XI (Q2QSR5), photosystem I reaction center subunit III, chloroplast (Q8S7H8), chlorophyll a-b binding protein, chloroplastic (Q5ZA98), glyceraldehyde-3-phosphate dehydrogenase (Q8H884), chloroplast photosystem I reaction center subunit II-like protein (Q84PB4), UPF0603 protein Os05g0401100, chloroplastic (Q6ATY4), ribulose bisphosphate carboxylase/oxygenase activase, chloroplastic (Q9LRH9) and photosystem I protein-like protein (Q84PB5) were hub proteins (Figure S8).

### 2.4. RT-qPCR-based Analysis of Target Genes and Validation of Transcriptome and Proteomic Data

**Figure 5:**
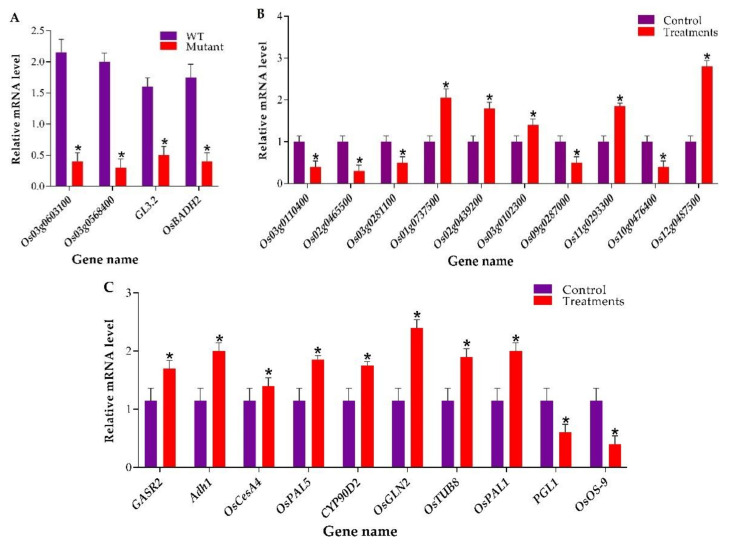
RT-qPCR-based assessment of target genes expression analysis and validation of transcriptome and proteomic experiments. (**A**) Expression level of *Os03g0603100*, *Os03g0568400*, *GL3.2*, and *OsBADH2* in wild-type (WT) and mutant plants (**B**) Expression analysis of ten selected DEGs and (**C**) genes associated with DEPs. * Denotes a significant difference, student’s *t*-test, *p* ≤ 0.01, *n* = 3.

RT-qPCR was performed to assess the expression level of target genes expression level in mutant plants and WT. The RT-qPCR results exhibited that the expression of *Os03g0603100*, *Os03g0568400*, *GL3.2,* and *OsBADH2* was significantly suppressed in mutant plants as compared to WT (Figure 5A). For the verification of transcriptome data, five upregulated DEGs (*Os01g0737500*, *Os02g0439200*, *Os03g0102300*, *Os11g0293300,* and *Os12g0487500*) and five downregulated DEGs (*Os03g0110400*, *Os02g0465500*, *Os03g0281100*, *Os10g0476400,* and *Os09g0287000*) were selected. The results clearly showed that the RT-qPCR based expression analysis of these genes showed a similar expression pattern to transcriptomic data (Figure 5B). To validate the proteomic data, we selected the ten genes associated with DEPs. In total, two key genes encoding downregulated proteins including *PGL1* and *OsOS-9*, and eight genes encoding upregulated proteins including, *GASR2, Adh1, OsCesA4, OsPAL5, CYP90D2, OsGLN2, OsTUB8,* and *OsPAL1,* were chosen. The results of RT-qPCR results revealed that the expression level of these genes was similar to the proteomic data (Figure 5C). The primers used for RT-qPCR are enlisted in Table S9.

## 3. Discussion

Significant improvement has been made in the study of rice yield improvement in the past two decades. The simultaneous advancement in genome editing methods, such as TALENs, ZFNs, and CRISPR/Cas9, has brought new potentials for the direct regulation of genes involved in rice yield. These achievements can be used either to improve crop yields or to elucidate the function of genes controlling grain yield. Currently, CRISPR/Cas9-based gene editing has been widely used to improve the yield in crop plants [15,16,17,18,34]. Crop improvement through genome editing provides decent prospects to create mutants with desired traits. However, examples to create novel genotypes and to yield and quality improvement together using SSNs are very few. The CRISPR/Cas9 system is widely used for targeted mutagenesis in many species because of its simplicity and high efficiency. In the present work, we used the CRISPR/Cas9 system to successfully edit four genes. The mutation efficiency of eight targets was found to be very high. Mutants are essential for crop breeding and gene research. However, the integration of excellent genes from different rice varieties into one breeding variety is facing challenges.

Output of conventional breeding became limited due to the genetic bottleneck created by the continuous selection process which restricts breeding productivity [47,48]. Natural selection in random or uncertain and unguided artificial mutagenesis results in a very limited frequency of mutational events at interested target loci [48]. Alternatively, CRISPR/Cas9 produces high-density populations of mutants in pre-decided target loci [49]. We applied RNA-sequencing and iTRAQ-based proteomic analysis to confirm the effects of CRISPR/Caas9 genome editing at gene and protein levels. The employment of advanced technologies together to elucidate the differences among mutant plants and WT provides understandings about the impacts of mutations on whole-genome level.

The main functional parts of CRISPR/Cas9 editing vector is RNA polymerase III (Pol III) promoter-driven gRNA expression cassette and 35S/ubiquitin promoter-driven Cas9 expression cassette. Due to the limiting transcriptional activity of Pol III promoter, the previous studies used small nuclear RNA promoters (U3 and U6) from rice. U3 and U6 promoters have definite transcription beginning sites A nucleotide and G nucleotide, respectively [50,51]. In this study, we also used U3 and U6 (OsU6a, OsU6b, OsU6c, and OsU3m) promoters to construct the expression cassette by selecting the target sequences with 5’-AN(19)NGG and 5’-GN(19)NGG, respectively. Following the cloning strategies, eight sgRNA were successfully ligated in expression cassette driven by U3 and U6 promoters for targeting four genes in rice.

The functions of Cyt P450 gene family have been extensively studied [30]. In rice, there are 356 Cyt P450 genes and 99 related pseudogenes, while in Arabidopsis there are 246 Cyt P450 genes and 26 pseudogenes existing approximately 200 million years ago. The multiplicity of the same family genes has hampered the study of different Cyt P450 individual genes. Early work revealed that P450 genes are involved in the plant metabolism, fatty acids, leukotrienes, pheromones, biogenic amines, prostaglandins, and various oxidative metabolism of steroids [47]; however, CRISPR knockout mutants have not been studied before.

The CRISPR/Cas9 construct transformation method has direct relationships with mutation type and frequencies. In our work, we obtained 40% of transformation and 57.32% mutation efficiency which is higher than many other previous reports. The efficiency and access of Cas9/sgRNA ribonucleoprotein’s is mainly affected by the chromosomal location, a sequence of the target gene and multiplex nature of sgRNAs.

### 3.1. CRISPR Mutants Exhibited Inheritable Mutations, Increased Grain Yield, and Fragrant Phenotype

In our study, the homozygous and heterozygous mutations were more frequent, while chimeric and bi-allelic mutations were rare. According to the previous studies, the compound heterozygous and homozygous mutations mostly occurred in T_0_ generation [52,53]. The homozygous mutant plants were obtained in T_0_ generation showing stable transmission of mutation to the subsequent T_1_, T_2_, and T_3_ generations. Homozygous mutant plants have been achieved in the T_0_ generation in previous studies [53].

To avoid the random mutations, specific of CRISPR/Cas9 also requires consideration during the genome editing experiments. Previous studies reveal that there is a low possibility of off-target mutations in rice crop [16,17,18], but to satisfy the biosafety concerns and rules, it is critical to evaluate and select the transgene-free mutant plants. We also followed the genetically modified (GM) regulations to avoid public controversy and social acceptance issues. In this study, we screened the T-DNA-free lines with a frequency of 44.44%. We also evaluated and verified the off-target free mutant plants by selecting the five most likely putative off-target sites against each target.

The segregation of genome modifications and predictable inheritance in offsprings is very important in basic research and molecular breeding. In the present study, it was observed that the Cas9-induced mutations were highly stable and inheritable to the subsequent generations in a similar pattern as unmutated loci follow. The segregation analysis in T_1_ generation showed that homozygous mutations were stably transmitted without any new mutations or inversion according to Mendelian law. Heterozygous and bi-allelic mutations followed the 1:2:1 segregation principle while the segregation of chimeric mutations was unpredicted. This concludes that Cas9 did not induce the new mutation in homozygous, heterozygous, and bi-allelic T-DNA-free mutants. The reason for the unpredicted segregation of chimeric mutants is unclear which needs further experiments on large mutant populations. Previous studies also suggest that the chimeric mutations could continue to mutate either in T_0_ or T_1_ generation [53,54].

In the present study, we produced the single, double, and triple mutants of Cyt P450 genes. The analysis of the phenotypes of mutant plants showed that three of the P450 genes are functional and mutations in these genes responsible for an increase in GWT. We generated CP450 and *OsBadh2* mutants, with increased GL, GWD, GWT, and 2AP content. To determine whether every gene has a functional role in the regulation of grain size, we recorded the data for GWT in T_0_ generation for each line and found that mutation in each P450 gene confers enhanced GWT. In comparison with WT the transgenic plants of *Os03g0603100*, *Os03g0568400* and *GL3.2* had a significant increase in GWT. The mutant plants with mutations in a single target site showed a non-significant difference or a slight increase in GWT. Plants with mutations in two genes had increased GWT, while their GWT was less than those of mutant plants with mutations in all target sites. The homozygous mutant plants for all target sites showed a 24.64% increase in GWT. These results suggest that these P450 genes have an individual effect on increased GWT. These results indicate that these genes regulate spikelet hull development. Rice *GIANT EMBRYO* (*GE*) encodes CYP78A13 and control cell death in endosperm and cell size in the embryo [55,56]. *CYP78A13* was found to be responsible for GWT, whereas its P450 paralogue *GL3.2*, is associated with GL [57], while their expression pattern differs. Microscopic analysis showed that the mutant showed a 23.23% increased cell number because of increased cell division. These results designated a connection between enhanced grain and cell numbers. Arabidopsis CYP78A5 mutants displayed small leaves due to decreased petal and leaf cells while there was no change was observed in cell size [58]. Moreover, Arabidopsis double-mutants (*cyp78a5* and *cyp78a7*) show higher leaf initiation rate, short petioles, small round rosette leaves, and small sterile flowers [59]. In rice, *DSS1* gene which belongs to Cyt P450 gene family is responsible for rice grain growth and drought tolerance [60]. The *PLA1* represents a similar function to *CYP78A7* and *CYP78A5* overexpressing Arabidopsis mutants showing larger flowers and leaves and strong apical dominance due to increased number of cells [59,61]. Moreover, *CYP78A9* and *CYP78A6* enhance seed growth by promoting the cell expansion of developing seeds integuments [27,62].

The grains of homozygous mutant lines and WT were assayed for 2AP level through GC-MS and it was observed that mutant plants showed 2AP level of 0.72–0.78 mg/kg while in WT it was absent. Aromatic rice verities preferred by consumers because of distinct aroma and agreeable flavor and considered as the best quality premium rice. In a previous study, the researcher has generated TALEN mutants and achieved 0.35–0.75 mg/kg of 2AP content in rice. One of the fragrant rice variety Daohuanxiang has 0.5–0.75 mg/kg of 2AP level [22].

### 3.2. Cross-Regulation of Genes and Proteins Related to Cell Cycle, Grain Development and Cytochrome P450

A total of 2122 DEGs (413 upregulated and 1709 downregulated) and 104 DEPs (84 upregulated and 20 downregulated) were identified in GXU7-1 versus WT comparison. We selected top twenty genes with an ID cutoff showing highest mean expression. We found that *Os03g0227700*;*CYP90B2* (Cyt P450 90B2), *Os08g0512600*;*cdc2Os3* (B-type plant-specific cyclin-dependent kinase), and *Os12g0274700*;*OsRBCS2* (small subunit of Rubisco) showed higher expression level, while, *Os12g0291100*;*OsRBCS3* (small subunit of Rubisco*)*, and photosystem II proteins *(psbB* and *Os08g0200300*) showed lower expression level in GXU7-1. In rice, *OsDWARF4* belongs to CYP90B1 family which is highly expressed in leaves and roots and regulated by brassinosteroid pathway. It has been found that *Osdwarf4* mutants showed enhanced yield even under dense planting and low fertilizer [63]. Cyclin-dependent protein kinase (CDK) plays a key role in regulating the eukaryotic cell cycle [64] and required for the G2/M phase of the cell cycle. *CDKB2* is involved in DNA damage response and showed increased expression level upon DNA damage [65]. *Os12g0274700*;*OsRBCS2* belongs to *RBCS* multigene family which encodes a small ribulose-1,5-diphosphate carboxylase/oxygenase (Rubisco) subunit. There are four genes (*OsRBCS2-5*) encoding small Rubisco subunits in rice leaves. These four *RBCS* genes contribute to the accumulation of total enzymes in Rubsico regardless of the growth stage and the inhibition of a single *RBCS* gene cannot be completely complementary to other *RBCS* genes [66].

We also identified some highly expressed proteins responsible for controlling grain yield in rice. The proteins including Q0J7P4 (Putative HAP3 subunit of the CCAAT box-binding transcription factor), A0A0K2JL42 (protein containing a placenta-specific 8 domain), Q6YUX0 (Transcription factor ILI5), B9F4Q9 (E3 ubiquitin-protein ligase GW2) and Q6ZIK5 (Growth-regulating factor 4) showed higher mean expression in GXU7-1 as compared to WT. *OsHAP3H* is a HAP3 subunit of the HAP complex and transcription factor containing “CCAAT box-binding protein”, which regulates rice yield [67,68,69]. *OsPCR1 (protein containing a placenta-specific 8 domain)* knockdown lines had lighter grains, shorter grain lengths, and decreased grain weight by 12%, while *OsPCR1* overexpressed plants had an 8% increase in grain weights and increased grain lengths [70]. *PGL2* (*POSITIVE REGULATOR OF GRAIN LENGTH 2*) is an antagonistic pair of basic helix-loop-helix (bHLH) protein gene which regulated grain length by increasing the cell length [71]. *GW2* controls grain width and weight in rice and encodes an unknown RING-type protein having E3 ubiquitin ligase activity. *GW2* loss-of-function mutants lead to increased cell numbers, large spikelet hull, higher grain filling rate, and enhanced grain yield [5]. *OsGRF4* (*growth-regulating factor 4*) is a transcription activator that Positively regulates grain size by promoting cell division and expansion, leading to increased grain length and width [72].

We found eight DEGs (seven downregulated and one upregulated) and two DEPs (one upregulated and one downregulated) related to cyt P450 family. In Arabidopsis, CP450 family genes have been shown to regulate flower and seed development [73]. Mutations in *KLUH*/*CYP78A5* resulted in decreased seed size and small flowers due to reduced cell proliferation [64]. *CYP78A10* in Arabidopsis, which is a homolog of rice *GE* gene responsible for heavier and bigger seeds suggesting an important role of P450 gene family in the regulation of seed size [56]. *dwarf11* (*d11*) gene encode CYP724B1 which is feedback-regulated by brassinosteroid and responsible for reduced seed length [74].

Four DEGs including Os01g0633100/GIF2 (Grain incomplete filling 2), Os02g0762600 (SG1-short grain1-related protein), Os09g0517600 (Regulation of grain size), and Os03g0254400/OspPLAIIIα (Patatin-related phospholipase A) were downregulated in GXU7-1. GIF2 showed lower grain weight due to the slower filling and endosperm cell showed obvious defects [75]. SG1 preferably expressed in roots and young panicles and encodes a protein with unknown function and its overexpression phenotype produced short grains [76]. *OspPLAIIIα* overexpression lines resulted in dwarfing, reduced mechanical strength and cellulose content, and shorter stems, roots, leaves, seeds, and ears, while *OspPLAIIIα* knockout plants have longer ears and seeds [77].

We found three upregulated DEPs including Q6YUX0 (positive regulator of grain length 2;PGL2), Q6ZIK5 (grain size on chromosome 2;GS2), Q6YYV8 (BAHD acyltransferase-like protein gene; SLG), and two downregulated DEPs including B9F4Q9 (E3 ubiquitin-protein ligase;GW2), and Q6AT90 (Transcription factor APG;APG) related to grain size and development. PGL2 is a typical bHLH proteins gene that positively regulates the rice grain length by increasing the cell length of the inner epidermis of lemma [78]. Candidate gene study showed that SLG is a BAHD acyltransferase-like protein gene mainly expressed in lamina joints and young tissues and an important regulator of grain size, ideal plant type, and brassinosteroid homeostasis [79]. It has been found that GW2 encodes an unknown RING-type protein with E3 ubiquitin ligase activity, and its loss-of-function results in enhanced grain width, weight, and yield by increasing cell numbers [5]. Studies revealed that APG is an interactive part of PGL1 and APG silencing produced increased grain size by increasing the cell length of inner epidermal lemma cells [78].

We found that three rice cyclin genes and cyclin-dependent protein kinase genes, twenty-five genes related to cyclin-like F-box domain-containing protein were differentially expressed in GXU7-1 versus WT comparison. We also identified that three proteins including Q0J4I1 (cyclin-dependent kinase B2-1; *CDKB2-1*), P29618 (cyclin-dependent kinase A-1; *cdc2Os-1*) and Q6YXH8 (cyclin-D4-1; *OscyCD*) were upregulated in GXU7-1. Cyclin-dependent kinases (CDKs) regulate a complicated and precise process of cell division and proliferation in plants, animals and yeasts. Phosphorylation/dephosphorylation by proteolysis, kinases/phosphatases, and binding and activation of cyclin regulates the activity of CDKs [80,81,82]. Cyclin binding regulates protein stability as well as contributes to the subcellular localization and substrate specificity of the complex [83,84]. CDKs interact with cyclin to play a role at a specific phase of cell cycle and their periodic fluctuation at transcriptional and translational levels mainly depends on the cell-stage-specific activity of their cognate CDK partners [85].

In GO analysis we found that the DEGs related to CC were mainly enriched in a membrane-enclosed lumen, organelle lumen, intracellular organelle lumen and nuclear part. Regarding BP one of the most important category was cellular aromatic compound metabolic process with 311 genes. The DEGs related to MF were only enriched in double-stranded DNA binding. KEGG pathway analysis showed that the DEGs were enriched in only ribosomal pathways, whereas, hub-gene analysis also found all ribosomal proteins. Endomembrane organelles such as lumen of the endoplasmic reticulum, lysosome or vacuoles, the Golgi apparatus and the membranes of other organelles are evolved from plasma membrane. These organelles play important role in proteins trafficking to the plastid by the endomembrane system and then transported to the vacuole via secretory pathway [86]. In plant cells, a rice α-amylase (αAmy3) displayed dual localization to extracellular compartments and plastids [87]. Aroma genes are most intriguing for rice breeders to produce aromatic rice varieties. Further study is needed to explore the identified genes in this study to find out the mechanisms underlying aroma formation. Ribosomal proteins play a significant role in protein synthesis, apoptosis, ribosome biogenesis, and cell growth [88]. In eukaryotes, ribosomal proteins are encoded by multiple copies of genes. In rice, 123 genes encode 34 large subunit proteins [89].

GO analysis for DEPs revealed that, most of the BP, CC, and MF processes were enriched in photosynthesis. The KEGG analysis showed that DEPs were only enriched in photosynthesis pathways. In hub-proteins network analysis, we also found that most of the DEPs were related to photosystem I and photosystem II. Molecular engineering approaches to modify the specific genes to produce photosynthesis-related enzymes have been attempted in many studies [90,91,92,93,94,95,96]. Many quantitative trait loci (QTLs) responsible for photosynthesis regulation has been found in rice [97,98,99], however, a few of them (*Carbon assimilation rate 8 and green for photosynthesis*) has been fine mapped. To enhance the rice production, improvements in the photosynthetic rates is the main focus for rice production, however, more study is needed to elucidate the molecular basis of photosynthesis for effective rice breeding using nucleases.

In the current study, we provided a case in which four genes were edited and achieved highly efficient mutagenesis frequency without the introduction of unwanted and deleterious mutations. Novel alleles generated in this study will provide ideal genetic material for crop breeding to rapid improvement of quality and yield of rice. In this study, the success of multiplex gene editing made us sure that the multiple genes can be edited simultaneously to improve the rice yield at a significant level. As shown in this study, the rice grain fragrance, GL, and GWD were improved, while the other traits were not affected. According to the findings in this study, we highly recommend that desirable rice material should be achieved by rationally editing those genes which control important traits in rice.

## 4. Material and Methods

### 4.1. Experimental Material and Growth Conditions

The rice variety IR-96 was used for mutagenesis and seeds were obtained from the Rice Research Institute of Guangxi University (RRI-GXU), Nanning, China. During the natural growing seasons, and the plants were cultivated in the experimental field of GXU. The WT plants obtained from non-transformed seed of the IR-96 variety were used as control with mutant lines for genotyping and agronomic data collection. The Cas9 vector (pYLCRISPR/Cas9Pubi-H; Addgene plasmid #66187) and gRNA promoters (OsU6a, OsU6b, OsU6c, and OsU3m) were obtained from Liu Yaoguang’s Lab, South China Agricultural University, Guangzhou, China. The pYLCRISPR/Cas9Pubi-H is a plant binary vector in which Cas9p was driven by the maize ubiquitin promoter (Pubi) with hygromycin selectable marker gene having two *BsaI* sites flanked by a toxic negative selectable marker (ccdB gene) for the cloning of sgRNA expression cassette (Figure S9A) [100].

### 4.2. SgRNAs Selection and Off-targets Prediction

Three Cyt P450 homoeologs [Cyt P450 78A11 (*Os03g0603100*), Cyt P450 71E1 (*Os03g0568400*)*, Grain Length3.2* (*GL3.2*; *Os03g0417700*)] and *betaine aldehyde dehydrogenase (OsBADH2; fgr; Os08g0424500*) were identified and selected from rice genome using the online database RiceData (http://www.ricedata.cn/) [101]. Target sequences confirming the G(N)_20_ GG or (N)_20_ GG template were selected in the coding region of all genes by using online tool CRISPR-GE (http://skl.scau.edu.cn/) [102] (Table S10). To confirm the targeting specificity, a blast search of the selected sgRNAs (single-guided RNAs) including PAM (protospacer adjacent motif) was made against the rice genome using online tool NCBI (http://blast.ncbi.nlm.nih.gov/Blast.cgi) [103]. The first and second target for *Os03g0603100* was designed in the coding region from 3–22 bp and 161–142 bp, respectively. Both targets for *Os03g0568400* were designed in the first exon of 265–246 bp and 423–442 bp regions. The first and second target for *GL3.2* were selected in the first exon which were located in 49–68 bp and 436–455 bp regions, respectively. The first target of *OsBADH2* was designed in the first exon (165–184 bp) and the second target in the second exon (475–456 bp) (Figure S9B). The potential targets were selected having at least two base differences with similar non-target sequences. The targets having five or more than five mismatches to the non-target regions in the PAM distal region were not used as targets. The secondary structures of all sgRNAs were developed using the program RNAfold WebServer (http://rna.tbi.univie.ac.at//cgi-bin/RNAWebSuite/RNAfold.cgi) [104] (Figure S10A–H). Five potential off-targets containing two or more nucleotide mismatches for each gene were predicted using the CRISPR-GE by choosing the PAM-type parameters based on the PAM type of the on-target sgRNA (Table S3). The genomic sequences of four target genes were amplified and aligned with the reference sequence. After sequencing the sgRNAs sequences were verified in rice cultivar (IR-96) to ensure that there is no polymorphism between the sgRNAs and gene sequence. The oligo sequences used in constructing the sgRNA vectors are listed in Table S1.

### 4.3. Preparation of Templates and Generation of sgRNA Expression Cassettes

Following the principle of endogenous CRISPR/Cas9-directed multiplex mutagenesis, the vector was developed by using eight sgRNAs according to Ma et al. (2015) [100], with some modifications. The universal primers (Pps-R/Pgs-Pps-8/Pgs-L) were synthesized by the Beijing Genomics Institute (BGI). The *Bsa*I activity and digestions efficiency of the plasmid DNA was confirmed by setting up a reaction containing, 1.5 μL 10X CutSmart buffer, 350 ng of plasmid DNA, 4.5 U *Bsa*I-HF and 5.5 μL of ddH_2_O. The reaction was incubated at 37 °C for 15 min and digestions was checked electrophoresis on 1.5% agarose gel. The expression cassette was constructed by using Golden Gate Assembly (GGA) cloning method with two rounds of PCRs. The *lacZ* reporter was used only for the first target to monitor the success of GGA process. For each target, different U3/U6 promoters were used to diminish probable intra-plasmid recombination in Agrobacterium and loss of specific target-sgRNA sequences. In the first round of PCR, the UF/RP and FP/gR-R chimeric primer sets were used. In first PCR, separate amplifications were performed and 20 μL reaction was set up in which 0.2 μL of U6a-T1, 0.4 μL of KOD-FX, 4.0 μL of 5X buffer, 1.6 μL of dNTPs (2.5 mM), 0.4 μL of UF (primer), 0.2 μL of gRT1, 0.4 μL of gR-R, 0.5 μL of U6a, and ddH_2_O for 10 sec at 94 °C, 15 sec at 58 °C, and 20 sec 68 °C with 26 cycles. The same reaction was set up for all expression cassettes by using specific promoters and targets. The PCR products of all samples was confirmed on a 1.5% agarose gel. For the second PCR (nested PCR), 1 μL of PCR product of all targets was taken and diluted with 9 μL of ddH_2_O. Using the product from first PCR, a nested PCR for each sgRNA cassette was set up with a total of 30 μL reaction mixture as follows: 0.3 μL of KOD-FX, 6.0 μL of 5X buffer, 2.4 μL of dNTPs (10 mM), 1.5 μL of Pps-R, 1.5 μL of Pgs-2, 1.0 μL of U6a-T1 expression cassette and 19.4 μL of ddH_2_O, for 1 min at 95 °C, 10 sec at 98 °C, 30 sec at 58 °C, 40 sec at 68 °C, and 1 min at 68 °C with 26 cycles. The same reaction was followed for all expression cassettes by using the nested position-specific primers for each cassette (Table S1). The PCR products of all samples from nested PCR was confirmed on a 1.2% agarose gel. An equal amount (15 μL) of PCR products of all expression cassettes was mixed and purified by using TaKaRA MiniBest DNA Fragment Purification Kit Ver. 4.0 to remove the DNA polymerase enzyme and residual primers.

### 4.4. Assembly of Multiplex sgRNA Expression Cassette into CRISPR/Cas9Pubi-H Binary Vector

A 15 μL restriction-ligation reaction was set up with 1.5 μL 10X CutSmart Buffer, 0.5 μL of BsaI-HF (10U), 1.5 μL of 10X T4 Buffer, 0.1 μL of T4 DNA ligase (40U), 0.8 20 μL of intact pYLCRISPR/Cas9 plasmid, 0.5 μL of pooled and purified sgRNA expression cassette and 10.1 μL of ddH_2_O. The reaction was incubated for 15 cycles without heated lid (37 °C for 5 min, 10 °C for 5 min, 20 °C for 5 min, and 37 °C for 5 min). The ligation status was confirmed by agarose gel (1.0%) electrophoresis.

### 4.5. Transformation of *Escherichia* coli and Confirmation of Ligated Products

The ligated product added to competent cells of *E. coli* DH5α and transformed by the heat shock method. The sample was hand-shaken slightly and kept in ice for 30 min. After that, the sample was kept in a water bath at 42 °C for 45 sec and again in ice for 2 min. Then 500 μL of Super Optimal broth with Catabolite repression (SOC) medium was added and kept in the chamber for 1 h at 200 rpm and 37 °C. The bacterial solution was spread on LB plate containing 25 mg/mL kanamycin for 14 h. A single colony was picked and mixed in 1000 μL of LB medium containing Kanamycin (1 μL). The sample was shaken at 180 rpm and 37 °C until it became milky. Simple PCR was performed by using specific primers (SPL1/SPR) and products confirming the required length were directly sequenced and all targets were confirmed.

### 4.6. Rice Transformation and On/Off-target Mutation Analysis

The constructs were transformed into *A. tumefaciens* EHA105 by electroporation and rice transformation was performed according to Hiei et al. (1994) [105], with some modifications. Transformed EHA105 was used to genetically transform the embryogenic callus, and hygromycin selection was performed to screen transformed plants. A small amount of engineered bacterial solution was streaked on Yeast Extract Beef (YEB) plate culture medium and incubated in the dark at 27 °C for 48 hrs. A single colony was picked and transferred to 20 mL YEB liquid culture medium at 27 °C for overnight. The 800μL of the bacterial solution was transferred to 20mL YEB liquid medium (containing 200 μM/L *Acetosyringone*), and shaken for 4 hrs. The vigorously growing embryogenic calli were taken into a sterile triangle flask containing bacterial solution and finally co-cultured in dark at 27 °C for 2–3 days. The co-cultured calli were rinsed with ddH_2_O for 3–4 times and again cultured in dark for 5–7 days. Callus screening was performed after transferring the pre-culture callus to the selection medium and continue to culture for 4–7 weeks. The resistance calli were selected and transferred to the differentiation medium. After the green spots of embryonic callus appeared, the rooting and shooting medium was applied. After 2–3 weeks, the small plantlets were transferred to glass tubes containing Murashige and Skoog (MS) medium (containing 1/4MS inorganic salt). After the appearance of new roots and normal plantlet growth, the concentration of inorganic salt was reduced to 1/10MS. The plantlets were transplanted to field after appearance of several new roots. The DNA of T_0_ plants was extracted by cetyl trimethylammonium bromide (CTAB) method and target-specific primers (PT1T2F/R, PT3T4F/R, PT5T6F/R, PT7T8F/R; Table S1) were used for genotyping. The amplified products were sequenced by the Sanger sequencing method. The sequencing results were decoded by the online tool DSDecodeM (http://skl.scau.edu.cn/dsdecode/) [106]. Five putative off-targets for each target were examined using specific primers (POT1F/R-POT40F/R; Table S11). The DNA of T_1_ and subsequent T_2_ generation (T-DNA and T-DNA-free) was also extracted for genotyping and the inheritance pattern was studied. The T-DNA-free lines in T_1_ generation were screened by using specific primers (Cas9-F/R and HPTF/R) and the amplified product was observed on 1% agarose gel. Those plants were considered as T-DNA-free in which neither Cas9 nor HPT were detectable. After screening of T-DNA-free plants, we selected homozygous mutant plants for further investigations. The PCR fragments of homozygous mutant plants were directly sequenced using the corresponding site-specific primers, and the transmission of mutations were studied in T_1_, T_2_, and T_3_ generations. Strict self-pollination was performed in every generation to test the targeted mutations. Segregation of T-DNA-free mutant lines in T_1_ generation was analyzed. To confirm the Mendelian inheritance pattern, we performed the chi-square test of goodness-of-fit.

### 4.7. Phenotyping and Biochemical Analysis

The data for sGWT was recorded in T_0_ generation for all mutant lines and non-transformed WT plants, while the data for other agronomic traits were recorded for homozygous mutant lines in T_1_, T_2_ and T_3_ generations, such as PH, GWD, GL, GWT, GPPP, SSR. The 2AP content were determined by gas chromatography-mass spectrometry (GC-MS) and the previously described protocol was followed [107]. Rice grains (0.5 g) were dehulled and placed at high speed shaker in a 2 mL centrifuge tube to milled it thoroughly. The rice flour was taken in a 5 mL jaw bottle and 2 mL of extraction buffer solution was added, and 2, 4, 6-trimethylpyridine was used as an internal standard. The sample was cooled to room temperature after keeping it at 80 °C for 3 h and centrifuged for 5 min at 13,800 *g*. The supernatant was taken into a sample bottle and GC-MS device (GC7890A-5975C MS; Agilent Technologies, Santa Clara, CA, USA) was used to measure 2AP. Calculations were performed according to Shan et al. (2015) [22].

### 4.8. Library Preparation for RNA-Sequencing

Total RNA was extracted from leaf samples and mixed with DNase I and the oligo (dT) magnetic beads were used to enrich mRNA. The cDNA was prepared from mRNA fragments and purified with magnetic beads. Sequence adaptors were ligated after end preparation and 30-end single nucleotide A (adenine) addition to the fragments. Phusion High-Fidelity DNA polymerase, universal PCR primers, and Index (X) Primer were used for PCR to amplify the fragments for library construction. The PCR product was purified with AMPure XP system according to manufacturer instructions and quality was assessed on the Agilent Bioanalyzer 2100 system. cBot Cluster Generation System using TruSeq PE Cluster Kit v3-cBot-HS (Illumia) was used for the clustering of the index-coded samples according to the manufacturer’s instructions and libraries were sequenced and 150 bp paired-end reads were generated on an Illumina Hiseq 4000 platform.

### 4.9. Analysis of RNA-Sequencing Data

Primary sequencing data (raw reads) in FASTQ format were processed through scripts written in-house and reads containing ploy-N, low-quality reads and adapter were removed to obtain clean reads. The clean reads were aligned to the reference genome and reads numbers mapped to each gene were counted using HISAT2 v2.1.0 and HTSeq v0.11.2, respectively. The FPKM (fragments per kilobase of transcript per million mapped fragments) of each gene was calculated based on the length of the gene and reads count mapped to this gene. The DEA (differential expression analysis) was performed using the DESeq2 R package (1.26.0) and differentially expressed genes (DEGs) were screened with fold change ≥ 1.2 and *p*-value < 0.05. Gene Ontology (GO) enrichment analysis of DEGs was performed through agriGO (http://bioinfo.cau.edu.cn/agriGO/) [108], and Kyoto Encyclopedia of Genes and Genomes (KEGG) pathways of DEGs were tested by using KOBAS v3.0 software.

### 4.10. Protein Extraction, Digestion, and Labelling

Protein extraction was performed from the same leaf samples used for RNA-sequencing analysis according to Wang et al. (2014) [109]. Leaf samples were ground in liquid nitrogen (LN_2_) and a lysis buffer containing 100 mM NH4HCO3(pH = 8), 6 M Urea, and 0.2% SDS was added and centrifuged at 12,000× *g* for 15 min at 4 °C. Protein concentration was measured by Bradford protein assay and extracts from each sample were reduced with 2mM DTT for 1 h at 56 °C, and subsequently alkylated with enough Iodoacetamide for 1 h at room temperature in the dark. Samples were mixed with the 4 times volume of precooled acetone by vortexing and incubated for at least 2 h at −20 °C. Precipitation was collected after centrifugation of samples and washed twice with cold acetone. The pellet was dissolved by dissolution buffer containing 0.1 M triethylammonium bicarbonate (TEAB, pH = 8.5) and 6 M urea. Protein concentration was determined again by the Bradford protein assay. From each sample, the 0.12 mg of protein was digested by Trypsin Gold (Promega) at 1:50 enzyme-to-substrate ratio at 37 °C for 16 h, and vacuum dried. Peptides were labeled with iTRAQ reagents using iTRAQ^®^ Reagent-8PLEX Multiplex Kit, Sigma, following the manufacturer’s instructions.

### 4.11. HPLC Fractionation and LC-MS/MS Analysis

The reversed-phase fractionation of iTRAQ labeled and mixed peptides was done using a C18 column (Waters BEH C18 4.6 × 250 mm, 5 μm) on a Rigol L3000 HPLC operating at 1 mL/min, the column oven was set as 50 °C. The elution of peptides was performed at 0.8 mL/min flow rate. Buffer A comprised of 10mM Ammonium acetate, pH10.0 and buffer B (B) with 10mM Ammonium acetate 90% v/v CAN, pH of 10.0. The following gradient was applied for separation: 100% buffer A for 40 min, 0–5% B for 3 min, 5–35% B for 30 min, 35–70% B for 10min, 70–75% B for 10 min, 75–100% B for 7min, 100% B for 15 min and 100% buffer A for 15min finally. The absorbance measurement was done at 214 nm. Twenty fractions collected and pooled to obtain ten fractions for each sample. The fractions were vacuum centrifuged individually and reconstituted by using 40 µL of 0.1% v/v trifluoroacetic acid. Peptides analyzed by the Easy-nLC 1000 HPLC system which was attached to an Orbitrap Elite mass spectrometer (Thermo Fisher Scientific, San Jose, CA, USA). Each sample then loaded on a Thermo Scientific EASY column, with an autosampler at 150 nL/min. The further experimental process was carried out according to the previously established procedure [103].

### 4.12. Proteomic Data Analysis

The proteomics data was analyzed by using Proteome Discoverer 2.1 (Thermo Fisher Scientific, San Jose, CA, USA) against *Oryza sativa* subsp. Indica (Rice) database (September 17, 2018; 40,869 entries) with default parameters. The annotation of the identified proteins performed by using GO database (http://www.geneontology.org/) [110] and KEGG database (http://www.genome.jp/kegg/pathway). Enriched GO terms were identified with Fisher’s Exact Test and the Cluster 3.0 software used to perform cluster analysis of the DAPs (differentially accumulated proteins). PPI was assessed by STRING (http://string-db.org/) [111] and top-ten hub-genes based on higher betweenness score, degree, and closeness were predicted by Cytoscape (version 3.7.2) plugin, Cytohubba.

### 4.13. Determination of Transcript Level of Target Genes and Omics Data Validation

Total RNA was isolated by using TaKaRa MiniBEST Plant RNA Extraction Kit following the manufacturer instructions. RT-qPCR did by using a Real-Time (Roche) LC480 PCR with a total volume of 10 µl containing 0.08 µM gene-specific primers, 0.3 µl of reverse-transcribed product and 5.0 µl of ChamQTM Universal SYBR qPCR Master Mix (Vazyme). Rice *Actin* gene used as internal control and primers used were designed by using an online tool (https://biodb.swu.edu.cn/qprimerdb/) [112] (Supplementary Materials File S1, Table S9) and the expression was calculated by using 2^−ΔΔ^CT (cycle threshold) method as explained previously [113].

### 4.14. Statistical Analysis

The agronomic data were analyzed using SPSS 16.0 Statistical Software Program. GraphPad Prism (version 7.0, GraphPad Software Inc., San Diego, CA, USA) was used to develop the graphs. The data was analyzed by a two-tailed Student’s t-test and presented as the mean ± SD. The data were considered as statistically significant at a *p*-value of < 0.05.

## 5. Conclusions

Generations of T-DNA-free homozygous mutant lines with increased grain yield and stable inheritable mutations without any off-target effects are of great value in crop breeding. In this work, the CRISPR/Cas9 system was successfully used to edit *Os03g0603100*, *Os03g0568400*, *GL3.2*, and *OsBADH2* individually and simultaneously with high mutation frequency. We generated rice mutants with higher grain fragrance and yield both that may be useful for rice breeding. This study is an example to improve grain yield and quality together via the CRISPR/Cas9 system. These results indicate that the Cyt P450 gene family provides the opportunity to improve grain size and yield and can be utilized when breeding new elite varieties. Moreover, the characterization of remarkable transcriptome and proteome responses of mutant plants provides new insights into rice grain development. Our work provided essential resources for identifying some candidate genes and proteins, and biological pathways that might be involved in rice yield and quality of rice. The targeted multiplex genome editing also facilitated pathway-level study for engineered multigenic rice mutants with enhanced grain yield. To overcome the barriers in conventional breeding to improve yield and quality, CRISPR/Cas9 could be a promising approach. These results provide an excellent molecular mechanism underlying the Cyt P450 genes and *OsBADH2* mutations. The homozygous mutant lines will provide source material for rice breeding programs for yield improvement. Further studies underlying the mechanism of cell signaling in response to genome editing are warranted.

## Figures and Tables

**Table 1:** The number of plants detected and mutation frequency in T_0_ generation.

Number of Plants	T1	T2	T3	T4	T5	T6	T7	T8
Total	36	36	36	36	36	36	36	36
Homozygous	6	5	6	7	4	5	6	7
Mono-allelic heterozygous	16	14	9	12	10	11	13	16
Bi-allelic heterozygous	2	-	1	2	1	-	1	-
Chimeric	1	2	-	-	-	2	1	-
Wild type	11	15	20	15	21	18	15	13

**Table 2:** Performance of wild-type and homozygous mutant plants for main agronomic traits in T_1_, T_2_, and T_3_ generations.

Generation	Line	PH (cm)	GWD (mm)	GL (mm)	GWT (g)	GNPP	SSR (%)
T_1_	WT	95.3 ± 3.2	3.5 ± 0.5	9.3 ± 0.4	28.1 ± 0.4	139 ± 9	91.2 ± 3.6
	GXU7-1	93.7 ± 1.2 ^ns^	4.4 ± 0.5 *	11.3 ± 0.5 *	34.1 ± 0.4 *	139 ± 10 ^ns^	90.5 ± 3.3 ^ns^
	GXU19-1	95.5 ± 2.2 ^ns^	4.1 ± 0.3 *	11.2 ± 0.5 *	34.1 ± 0.3 *	141 ± 10 ^ns^	92.3 ± 4.3 ^ns^
	GXU24-1	97.8 ± 2.1 ^ns^	4.2 ± 0.2 *	11.4 ± 0.3 *	34.4 ± 0.4 *	143 ± 7 ^ns^	91.6 ± 4.3 ^ns^
	GXU28-1	96.3 ± 1.7 ^ns^	4.3 ± 0.4 *	11.2 ± 0.4 *	34.2 ± 0.3 *	141 ± 8 ^ns^	91.4 ± 4.3 ^ns^
T_2_	WT	94.9 ± 2.3	3.4 ± 0.4	9.4 ± 0.5	29.3 ± 0.3	140 ± 8	90.5 ± 4.4
	GXU7-2	96.7 ± 1.2 ^ns^	4.4 ± 0.3 *	11.3 ± 0.4 *	34.4 ± 0.4 *	141 ± 9 ^ns^	88.9 ± 3.5 ^ns^
	GXU19-2	95.6 ± 2.2 ^ns^	4.3 ± 0.4 *	11.4 ± 0.8 *	34.0 ± 0.4 *	140 ± 8 ^ns^	91.2 ± 3.2 ^ns^
	GXU24-2	97.5 ± 1.1 ^ns^	4.1 ± 0.3 *	11.3 ± 0.5 *	34.7 ± 0.3 *	141 ± 7 ^ns^	92.1 ± 4.3 ^ns^
	GXU28-2	96.5 ± 1.7 ^ns^	4.0 ± 0.5 *	11.5 ± 0.5 *	34.8 ± 0.3 *	139 ± 6 ^ns^	90.4 ± 2.3 ^ns^
T_3_	WT	97.5 ± 2.3	3.6 ± 0.4	9.2 ± 0.4	28.1 ± 0.3	143 ± 7	92.5 ± 4.5
	GXU7-3	94.8 ± 3.2 ^ns^	4.2 ± 0.3 *	11.2 ± 0.6 *	34.6 ± 0.4 *	142 ± 9 ^ns^	91.5 ± 3.3 ^ns^
	GXU19-3	95.6 ± 3.1 ^ns^	4.4 ± 0.3 *	11.4 ± 0.8 *	33.9 ± 0.4 *	139 ± 11 ^ns^	92.3 ± 5.3 ^ns^
	GXU24-3	96.4 ± 3.1 ^ns^	4.3 ± 0.5 *	11.3 ± 0.5 *	34.5 ± 0.4 *	140 ± 10 ^ns^	90.6 ± 3.3 ^ns^
	GXU28-3	96.3 ± 2.9 ^ns^	4.2 ± 0.5 *	11.5 ± 0.3 *	34.8 ± 0.3 *	140 ± 9 ^ns^	90.4 ± 5.3 ^ns^

WT: wild type; PH: plant height; GWD: grain width; GL: grain length; GWT; 1000 grain weight; GNPP: grain number per plant; SSR: seed setting rate. * and ^ns^ represent a significant and non-significant difference, respectively. Student’s *t*-test, *p* ≤ 0.01. These data are from the individual plant of the T_0_ generation and the mean of five independent samples from T_1_, T_2_, and T_3_ generations.

## Supplementary Materials

The following are available online at https://www.mdpi.com/2223-7747/9/6/788/s1, Figure S1. Sequence alignment of the (A) *Os03g0603100*, and (B) *Os03g0568400* gene in the reference genome and IR-96 rice variety. The SNPs between the reference genome and IR-96 are indicated with disappeared asterisks or dashes. The reference genome was used from Rice Genome Annotation Project website (http://rice.plantbiology.msu.edu/index.shtml); Figure S2: Transmission of mutations to subsequent generations in homozygous mutant plants; Figure S3: Transcriptome analysis of the wild type (WT) and mutant line (GXU7-1). (A) Box and whisker plot showing the plot for transformed read counts from transcriptome data of WT and GXU7-1; (B) Count data distribution frequency of transformed data from WT and mutant line; (C) Venn diagram showing the upregulated and downregulated differentially expressed genes (DEGs); (D) Heatmap of twenty most variable genes with higher mean expression values. Yellow color indicates gene IDs with a higher expression level and darker blue color represents a low expression level; Figure S4: Gene Ontology (GO) and Kyoto Encyclopedia of Genes and Genomes (KEGG) pathway-analysis of differentially expressed genes. (A) Histogram showing GO terms and KEGG pathway, BP; biological processes, CC; cellular component, and MF; molecular functions; (B) A diagram showing ribosome pathway. Red highlighted genes were upregulated, and those with white background indicate that the genes were not significantly up- or downregulated in GXU7-1 compared with WT; Figure S5: Top ten hub-genes selected from total differentially expressed genes (DEGs). Nodes represent genes, and edges indicate connections between the nodes. The redder color indicates a higher co-expression level; Figure S6: Basic information about proteomic analysis of the wild type (WT) and mutant line (GXU7-1). (A) Box-and-whisker plot of transcriptome count data distribution with log_2_ values of the normalized abundance; (B) Histogram for count data distribution frequency of transformed data from WT and mutant line; (C) Venn diagram showing the upregulated and downregulated differentially expressed proteins (DEPs); (D) Heatmap showing twenty DEPs with highest mean expression level. Yellow color indicates a higher expression level and dark blue color indicates lower expression level; Figure S7: Ontology (GO) and Kyoto Encyclopedia of Genes and Genomes (KEGG) pathway-analysis, BP; biological processes, CC; cellular component, and MF; molecular functions; (A) Histogram showing GO terms and KEGG pathways; (B) A diagram representing light-harvesting chlorophyll complex pathway. Enzymes represented red stars indicate that the corresponding proteins were upregulated, and other proteins were not significantly up- or downregulated in GXU7-1 compared with WT; Figure S8: The top 10 hub-proteins positively correlated with wild type (WT) and CRISPR/Cas9 mutant (GXU7-1). The red color denotes the higher co-expression; Figure S9: The map of vector and structure of four genes with positions of target sites. (A) Physical map of pYLCRISPR/Cas9Pubi-H vector. The CRISPR/Cas9 binary vector is approximately 16.5 kb in size and carries the resistance gene for hygromycin. It has left (LB) and right borders (RB), and from left to right, the *HPT* gene, the *Cas*9 driven by the ubiquitin promoter (P_ubi_), the NOS terminator (Tnos), and the *ccdB E. coli* lethal gene. When assembling the target, restriction endonuclease *Asc* I or *Bsa* I can be used to produce distinct, non-palindromic sticky ends. Single guided RNA (sgRNA) is followed by the U3/U6 promoters for genome targeting; (B) Gene structure and gRNA positions of *Os03g0603100*, *Os03g0568400*, *GL3.2,* and *OsBADH2.* T1 and T2 represent Target1 and Target2, respectively; Figure S10: Schematic representation of secondary structures of (A) sgRNA1; (B) sgRNA2; (C) sgRNA3; (D) sgRNA4; (E) sgRNA5; (F) sgRNA6; (G) sgRNA7; and (H) sgRNA8 in this experiment. The sgRNA structures shown above are colored by base-pairing probabilities. For unpaired regions, the color denotes the probability of being unpaired; Table S1: List of primers used for vector construction and genotyping of mutant plants; Table S2: Mutation types in T_0_ generation obtained by eight CRISPR/Cas9 constructs; Table S3: Detection of mutations on putative off-target sites; Table S4: Performance of 1000-grain weight (g) in T_0_ generation; Table S5: Segregation of CRISPR/Cas9-induced mutations in target genes; Table S6: Details about sequencing reads and quality output; Table S7: Differentially expressed genes (DEGs) related to cytochrome P450 family, grain development and cell cycle; Table S8: Differentially expressed proteins (DEPs) related to cytochrome P450, grain size and cell division; Table S9: Primers used for RT-qPCR analysis; Table S10: Target sites with their positions, GC content and potential off-target score; Table S11: Primers used for off-target analysis.
