# Supplementary material for: Generation of High Yielding and Fragrant Rice (Oryza sativa L.) Lines by CRISPR/Cas9 Targeted Mutagenesis of Three Homoeologs of Cytochrome P450 Gene Family and OsBADH2 and Transcriptome and Proteome Profiling of Revealed Changes Triggered by Mutations
Source: Plants (Basel). 2020 Jun 23;9(6):788. doi: 10.3390/plants9060788 (PMC7355857; doi:10.3390/plants9060788)
Supplement: Supplementary file 1 [file plants-09-00788-s001.zip › plants-818939 - supplementary for XML.pdf]

**Table S1.** List of primers used for vector construction and genotyping of mutant plants.

| Primer name | Primer sequence (5'-3') |
|---|---|
| PT1T2 | F: GGGTGAGAAGAAAGTGTGGGAT<br>R: TCTCCTGACACCACCCAACAT |
| PT3T4 | F: TCTCAACTAAACCACCACA<br>R: ACTCCTCCCTCTT |
| PT5T6 | F: CTCTTTTTCCCTGCTTTTT<br>R: GAAGAGGTGGTTGGCG |
| PT7T8 | F: CACTCCACACCTGACACCAC<br>R: TCCGATGCCGACGCACGCAC |
| gRT1 | ATCTTCCGTGGCACAGACAgttttagagctagaaat |
| OsU6aT1 | TGTCTGTGCCACGGAAGATCggcagccaagccagca |
| gRT2 | TTGACGACGCAGTGCAGGTAgttttagagctagaaat |
| OsU6aT2 | TACCTGCACTGCGTCGTCAACggcagccaagccagca |
| gRT3 | GGACCGGCTACAGCACTCGgttttagagctagaaa |
| OsU6bT3 | CGAGTGCTGTAGCCGGTCCCaacacaagcggcagc |
| gRT4 | CGATCGCCAGCGATCGCGCTgttttagagctagaaat |
| OsU6bT4 | AGCGCGATCGCTGGCGATCGCaacacaagcggcagc |
| gRT5 | CCATCTCCAGCGACATGTTCgttttagagctagaaat |
| OsU6cT5 | GAACATGTCGCTGGAGATGGCtgagcctcagcgcag |
| gRT6 | AGGCGCGCCCACGACAGGAGgttttagagctagaaat |
| OsU6cT6 | CTCCTGTCGTGGGCGCGCCTCtgagcctcagcgcag |
| gRT7 | TGGCCACGGCGATCCCGCAGgttttagagctagaaat |
| OsU3T7 | CTGCGGGATCGCCGTGGCCATgccacggatcatctgc |
| gRT8 | CACGGCGGAGGACGTGGACGgttttagagctagaaat |
| OsU3T8 | CGTCCACGTCCTCCGCCGTGTgccacggatcatctgc |
| U-F | CTCCGTTTTACCTGTGGAATCG |
| gR-R | CGGAGGAAAATTCCATCCAC |
| Pps-R | TTCAGAggtctcTACCGACTAGTATGGAATCGGCAGCAAAGG |
| Pgs-2 | AGCGTGggtctcGtcagggTCCATCCACTCCAAGCTC |
| Pps-2 | TTCAGAggtctcTctgacacTGGAATCGGCAGCAAAGG |
| Pgs-3 | AGCGTGggtctcGtcttcacTCCATCCACTCCAAGCTC |
| Pps-3 | TTCAGAggtctcTaagacttTGGAATCGGCAGCAAAGG |
| Pgs-4 | AGCGTGggtctcGagtccttTCCATCCACTCCAAGCTC |
| Pps-4 | TTCAGAggtctcTgactacaTGGAATCGGCAGCAAAGG |
| Pgs-5 | AGCGTGggtctcGgtccacaTCCATCCACTCCAAGCTC |
| Pps-5 | TTCAGAggtctcTggacttgTGGAATCGGCAGCAAAGG |
| Pgs-6 | AGCGTGggtctcGcagatagTCCATCCACTCCAAGCTC |
| Pps-6 | TTCAGAggtctcTtctgcaaTGGAATCGGCAGCAAAGG |
| Pgs-7 | AGCGTGggtctcGacctcaaTCCATCCACTCCAAGCTC |
| Pps-7 | TTCAGAggtctcTaggtttcTGGAATCGGCAGCAAAGG |
| Pgs-8 | AGCGTGGGTCTCGAGCGTTCTCCATCCACTCCAAGCTC |
| Pps-8 | TTCAGAGGTCTCTCGCTGATTGGAATCGGCAGCAAAGG |
| Pgs-L | AGCGTGGGTCTCGTCTTCACTCCATCCACTCCAAGCTC |
| PB-R | GCGCGCGGTCTCTACCGACGCGTATCC |
| PB-L | GCGCGCgGTCTCGCTCGACTAGTATGG |
| HPT | F: GTGCTTGACATTGGGGAGTT<br>R: ATTTGTGTACGCCCGACAGT |
| Actin-F | GAGTATGATGAGTCGGGTCCAG |
| Actin-R | ACACCAACAATCCCAAACAGAG |
| Cas9-F | CTGACGCTAACCTCGACAAG |

| | |
|---|---|
| Cas9-R | CCGATCTAGTAACATAGATGACACC |
| SP-L1 | GCGGTGTCATCTATGTTACTAG |
| SP-R | GCCTATACCAAGTTATTGCA |

**Table S2.** Mutation types in $T_0$ generation obtained by eight CRISPR/Cas9 constructs.

| Lines | T1 | T2 | T3 | T4 | T5 | T6 | T7 | T8 |
|---|---|---|---|---|---|---|---|---|
| GXU1 | 1i/2d | 2i/2d | WT | 3d/2d | 1d/3d | WT | 3d/2d | 4d/3i |
| GXU2 | WT | 1d/- | 1d/1i | WT | 10d/10d | 3d/2i | WT | WT |
| GXU3 | 6d/- | WT | 1d1i/- | 2d/- | 4d/- | WT | 2d/- | 3d/- |
| GXU4 | 2d/2d | 2d/- | WT | WT | WT | 2d/- | WT | WT |
| GXU5 | WT | 2d/- | WT | 1d/- | WT | 3d/- | 2d/- | 1d/- |
| GXU6 | 2d/- | WT | WT | WT | WT | WT | WT | WT |
| GXU7 | 6d/6d | 7d/7d | 2d/2d | 4d/4d | 1d1i/1d1i | 2d/2d | 8d/8d | 5d/5d |
| GXU8 | 4d/- | WT | WT | 1d/- | WT | WT | WT | 2d/- |
| GXU9 | 4d/- | 1d/- | 2d/- | 3d/3d | 1d/- | 3d/- | WT | 2d/- |
| GXU10 | WT | WT | WT | 2d1i/- | WT | WT | 1i/- | WT |
| GXU11 | 1d/- | 1d/2d | WT | WT | WT | 3d/- | WT | 1d/1d |
| GXU12 | 2d/- | WT | 1d/- | WT | WT | WT | WT | 2d/- |
| GXU13 | 1i/- | 1i/- | WT | 1d/- | 3i/- | WT | 2d/2d | 3d/- |
| GXU14 | WT | 3d/- | 3d/- | WT | WT | 1d/1d | WT | WT |
| GXU15 | 14d/- | WT | WT | 10d/10d | 12d/12d | WT | WT | 1d/- |
| GXU16 | 1d/1d | 1d/- | WT | WT | WT | 3d/- | 3d/- | WT |
| GXU17 | 2d/- | WT | 12d1i/- | 2d/- | 1d/- | WT | WT | 2d/- |
| GXU18 | WT | WT | WT | WT | WT | WT | 2i/- | 2d/- |
| GXU19 | 8d/8d | 5d/5d | 2d/2d | 3d/3d | 10d/10d | 7d/7d | 2d/2d | 6d/6d |
| GXU20 | 1d/- | 1d/- | WT | 1d/- | WT | WT | WT | WT |
| GXU21 | 1i/- | WT | 1d/- | WT | WT | WT | 2d/- | 3d/- |
| GXU22 | WT | 1i/- | WT | 1d/- | 2d/- | WT | WT | 3d/- |
| GXU23 | 12d/- | WT | 1d/- | WT | WT | WT | 2i/- | WT |
| GXU24 | 2d/2d | 4d/4d | 1d/1d | 2d1i/2d1i | 5d/5d | 4d/4d | 6d/6d | 2d/2d |
| GXU25 | WT | WT | WT | 1i/- | WT | WT | WT | WT |
| GXU26 | 1i/1i | 1d/- | WT | WT | 2d/- | 3d/- | 2d/- | 3d/- |
| GXU27 | WT | 2d/- | 2d2i/- | WT | WT | WT | WT | WT |
| GXU28 | 1d/1d | 1d/1d | 15d/15d | 12d1i/12d1i | 7d/7d | 7d/7d | 6d/6d | 3d/3d |
| GXU29 | 2i/- | 1d2i/- | WT | 1i/- | WT | WT | WT | WT |
| GXU30 | 1d/2i | 1i/1i | 1d/- | WT | WT | 2d/- | 2i/- | 1d/- |
| GXU31 | WT | 1d/- | 1d/1d | 1d/- | 2d/- | 2d/- | 4d/4d | 1i/1i |
| GXU32 | 1d/- | 2d/- | WT | WT | 1i/- | 1i/- | 2d/1i | 2d/- |
| GXU33 | 1i/- | 3d/- | WT | 1d/- | WT | 1d/2d | 1i/- | 1d1i/1d1i |
| GXU34 | WT | 1d/1d | 1d/- | 5d/- | 3d/- | WT | 2d/- | WT |
| GXU35 | 1d/- | 1d/1i | WT | 1i/5d | WT | 1i/- | 2d/- | 2d/- |
| GXU36 | WT | 2i/- | WT | WT | 3d/- | 2d/- | 3d/- | WT |

T: target, d: deletion, i: insertion and WT: wild type (no mutation). The numbers in front of the letters indicate the number of nucleotides deleted or inserted. Corresponding mutations in two alleles are distinguished by '/'.

**Table S3.** Detection of mutations on putative off-target sites.

| Target | NOPO | GL | Locus | Sequence | MMB | NPS | NPM | OTS | Region |
|---|---|---|---|---|---|---|---|---|---|
| T1 | OT1 | Chr6:26154498 | *LOC_Os06g43500* | CCTCTTGCGTGGCACAGGCACGG | 4 | 30 | 0 | 0.106 | CDS |
| | OT2 | Chr4:25806356 | *LOC_Os04g43610* | GAGCTGGCGTGGGACAGACAAGG | 4 | 30 | 0 | 0.018 | intron |
| | OT3 | Chr8:27421582 | *LOC_Os08g43390* | GATCTTTCGAGGGACGGACACGG | 4 | 30 | 0 | 0.018 | CDS |
| | OT4 | Chr6:8986787 | *LOC_Os06g15830* | GATCTTTAGAGGGACGGACACGG | 5 | 30 | 0 | 0.012 | CDS |
| | OT5 | Chr1:4405273 | *LOC_Os01g08780* | GCTCTTCCCTGGCACCGATACGG | 4 | 30 | 0 | 0.000 | CDS |
| T2 | OT6 | Chr1:15582450 | *LOC_Os01g27890* | TTGATGACGAGGTGCAGGTAGGG | 3 | 30 | 0 | 0.358 | CDS |
| | OT7 | Chr7:22544654 | *LOC_Os07g37620* | TTGATGACGAGGTACAGGTACGG | 4 | 30 | 0 | 0.269 | CDS |
| | OT8 | Chr4:401260 | *LOC_Os04g01600* | CTGACGACGCAGCGCAGGGAAGG | 3 | 30 | 0 | 0.169 | CDS |
| | OT9 | Chr1:15980235 | *LOC_Os01g28540* | ATGGCGACGCAGTGCAGGTACAG | 3 | 30 | 0 | 0.162 | CDS |
| | OT10 | Chr6:20302483 | *LOC_Os06g34910* | TTGAGGACGATGTGCAGGTAGGG | 3 | 30 | 0 | 0.160 | CDS |
| T3 | OT11 | Chr1:5739389 | *LOC_Os01g10720* | GGGACTGGCTTGAGCAATGGGGG | 5 | 30 | 0 | 0.007 | CDS |
| | OT12 | Chr11:7392211 | *LOC_Os11g13490* | TGCACCGGCTGCAGTACTCCGGG | 5 | 30 | 0 | 0.007 | CDS |
| | OT13 | Chr2:10086389 | *LOC_Os02g17520* | TGGACTGGCTGCCGCACTCGGTG | 5 | 30 | 0 | 0.005 | CDS |
| | OT14 | Chr4:25646231 | *LOC_Os04g43370* | GCGACCGGCAACAGACCACGGGG | 5 | 30 | 0 | 0.000 | CDS |
| | OT15 | Chr4:29126793 | *LOC_Os04g48840* | GGGACGGGCCACAGCACTGGGAC | 5 | 30 | 0 | 0.000 | CDS |
| T4 | OT16 | Chr8:8662935 | | CGATCGCCAGCGATCGCGCTGGG | 2 | 30 | 0 | 0.289 | intergenic |
| | OT17 | Chr1:21744556 | | AGATCGCCATCGATCGCGCTGGG | 2 | 30 | 0 | 0.813 | intergenic |
| | OT18 | Chr5:26916915 | *LOC_Os05g46460* | CGATCGCCGGCAAAGGCGATGGG | 5 | 30 | 0 | 0.004 | intron |
| | OT19 | Chr1:25949884 | *LOC_Os01g45700* | CGATCGGCAGCAATTGCAAGGTG | 5 | 30 | 0 | 0.000 | 3′UTR |
| | OT20 | Chr10:3612274 | *LOC_Os10g06930* | CGATCGCCAGCAAGCACCCGGCG | 5 | 30 | 0 | 0.000 | CDS |
| T5 | OT21 | Chr8: 27252257 | *LOC_Os04g46000* | ACGTCTCCGGCGACACGTTCAGG | 4 | 30 | 0 | 0.272 | CDS |
| | OT22 | Chr11: 10374014 | *LOC_Os11g18366* | ACGTCTCCAGCGGCACGTTCAGG | 4 | 30 | 0 | 0.266 | intron |
| | OT23 | Chr5: 18694268 | *LOC_Os05g32070* | CCATCAACGGCCACATGTTCAGG | 4 | 30 | 0 | 0.229 | CDS |
| | OT24 | Chr10: 12264294 | *LOC_Os10g23910* | ACATCCCAAGCGACATGGTCAGG | 4 | 30 | 0 | 0.197 | intron |
| | OT25 | Chr1: 36938702 | *LOC_Os01g63680* | CCGGCGCCAGTGACATGTTCAGG | 4 | 30 | 0 | 0.067 | CDS |
| T6 | OT26 | Chr2: 14391885 | *LOC_Os02g24800* | AGGCGCGACCACGACAGGAGACG | 2 | 30 | 0 | 0.070 | CDS |
| | OT27 | Chr10: 1717447 | *LOC_Os10g03800* | AGGTGCACCCGCGGCAGGAGCGG | 4 | 30 | 0 | 0.243 | CDS |
| | OT28 | Chr4: 7033034 | *LOC_Os04g12720* | CGGCGCGCCCACGGCAGGTGCGG | 3 | 30 | 0 | 0.215 | CDS |
| | OT29 | Chr11: 682575 | *LOC_Os11g02340* | AGGCCCGCCCAATACAGGAGCGT | 4 | 30 | 0 | 0.215 | CDS |
| | OT30 | Chr2: 18639857 | *LOC_Os02g31140* | TGGCGCTCGCACGACAGCAGCGG | 4 | 30 | 0 | 0.168 | CDS |
| T7 | OT31 | Chr11:23845456 | *LOC_Os11g39990* | CGGCGGCCGCTCTTCGCCGCCGG | 3 | 30 | 0 | 0.242 | CDS |
| | OT32 | Chr5:10796729 | *LOC_Os05g18640* | CCGCGCCAGCGCTTCGTCGCCGG | 3 | 30 | 0 | 0.214 | CDS |
| | OT33 | Chr4:18948998 | *LOC_Os04g31630* | CAGCGGCAGAACGTCGTCGCCGG | 3 | 30 | 0 | 0.170 | CDS |
| | OT34 | Chr2:7959861 | *LOC_Os02g14460* | CAGCGGCAGCGCGTCGTCGCCGG | 2 | 30 | 0 | 0.104 | CDS |

| | | | | | | | | | |
|---|---|---|---|---|---|---|---|---|---|
| | OT35 | Chr6:1048283 | *LOC_Os06g02850* | CAGGGGCAGCTCGGCGTCGGCGG | 4 | 30 | 0 | 0.000 | CDS |
| T8 | OT36 | Chr1:25206580 | *LOC_Os01g43980* | TCCTTCTCCTGGACGGCGCCCGG | 4 | 30 | 0 | 0.288 | CDS |
| | OT37 | Chr5:16461459 | *LOC_Os05g28140* | ATCTTCTCCCGGACGGCGCCCGG | 4 | 30 | 0 | 0.283 | CDS |
| | OT38 | Chr5:16168108 | *LOC_Os05g27760* | ATCTTCTCCCGGACGGCGCCTGG | 4 | 30 | 0 | 0.283 | CDS |
| | OT39 | Chr4:23175872 | *LOC_Os04g39020* | TACTTGGCCCGCACGGCCCCAGG | 2 | 30 | 0 | 0.252 | CDS |
| | OT40 | Chr3:30886096 | *LOC_Os03g53860* | AAGTTGGCCGGGACGTCGCCCTG | 5 | 30 | 0 | 0.000 | CDS |

Note: The protospacer adjacent motif (PAM) (NGG) is shown in green background. NOPO; name of putative off-target, GL; genomic location, MMB; mismatching bases, NPS; number of plants screened, NPOM; number of plants with off-target mutations, OTS; off-target score. T1-T8 represents target1 to target8.

**Table S4.** Performance of 1000-grain weight (g) in $T_0$ generation.

| Genotype | GWT (g) | Genotype | GWT (g) | Genotype | GWT (g) |
|---|---|---|---|---|---|
| WT | 28.0 ± 0.4 | GXU13 | 33.9 ± 0.3* | GXU26 | 33.5 ± 0.3* |
| GXU1 | 31.8 ± 0.1* | GXU14 | 32.9 ± 0.2* | GXU27 | 28.9 ± 0.5$^{ns}$ |
| GXU2 | 31.9 ± 0.3* | GXU15 | 33.9 ± 0.4* | GXU28 | 34.9 ± 0.3* |
| GXU3 | 32.1 ± 0.2* | GXU16 | 33.8 ± 0.5* | GXU29 | 33.8 ± 0.3* |
| GXU4 | 29.1 ± 0.3$^{ns}$ | GXU17 | 34.0 ± 0.5* | GXU30 | 34.1 ± 0.4* |
| GXU5 | 31.2 ± 0.2* | GXU18 | 33.7 ± 0.3* | GXU31 | 33.2 ± 0.2* |
| GXU6 | 28.6 ± 0.3 $^{ns}$ | GXU19 | 33.9 ± 0.4* | GXU32 | 31.2 ± 0.5* |
| GXU7 | 34.5 ± 0.5* | GXU20 | 32.9 ± 0.5* | GXU33 | 32.1 ± 0.2* |
| GXU8 | 29.1 ± 0.4$^{ns}$ | GXU21 | 33.2 ± 0.4* | GXU34 | 33.5 ± 0.6* |
| GXU9 | 33.2 ± 0.4* | GXU22 | 33.6 ± 0.5* | GXU35 | 32.5 ± 0.2* |
| GXU10 | 33.6 ± 0.1* | GXU23 | 33.3 ± 0.2* | GXU36 | 33.8 ± 0.5* |
| GXU11 | 31.5 ± 0.4* | GXU24 | 34.6 ± 0.4* | | |
| GXU12 | 34.2 ± 0.2* | GXU25 | 28.8 ± 0.3$^{ns}$ | | |

* and ns represent a significant and non-significant difference, respectively. Student's t-test, $p \leq 0.01$.

**Table S5.** Segregation of CRISPR/Cas9-induced mutations in target genes.

| Targets | $T_0$ | | | $T_1$ | | | | |
|---|---|---|---|---|---|---|---|---|
| | Mutants | Zygosity | InDels | PT | WT | Bi | Homo | Hetero |
| T1 | GXU7-1 | Hom | 6d/6d | 15 | 0 | 0 | 15(6d/6d) | 0 |
| T2 | GXU7-1 | Hom | 7d/7d | 15 | 0 | 0 | 15(6d/6d) | 0 |
| T3 | GXU7-1 | Hom | 2d/2d | 15 | 0 | 0 | 15(6d/6d) | 0 |
| T4 | GXU7-1 | Hom | 4d/4d | 15 | 0 | 0 | 15(6d/6d) | 0 |
| T5 | GXU7-1 | Hom | 1d1i/1d1i/ | 15 | 0 | 0 | 15(6d/6d) | 0 |
| T6 | GXU7-1 | Hom | 2d/2d | 15 | 0 | 0 | 15(6d/6d) | 0 |
| T7 | GXU7-1 | Hom | 8d/8d | 15 | 0 | 0 | 15(6d/6d) | 0 |
| T8 | GXU7-1 | Hom | 5d/5d | 15 | 0 | 0 | 15(6d/6d) | 0 |
| T1 | GXU1-1 | Het | 1i/2d | 42 | 11 | 0 | 21(1i/1i) | 10(1i/2d) |
| T2 | GXU1-1 | Het | 2i/2d | 42 | 13 | 0 | 20(2d/2d) | 9(2i/2d) |
| T3 | GXU2-1 | Het | 1d/1i | 42 | 11 | 0 | 20(1i/1i) | 11(1d/1i) |
| T4 | GXU1-1 | Het | 3d/2d | 42 | 12 | 0 | 21(2d/2d) | 9(3d/2d) |
| T5 | GXU1-1 | Het | 1d/3d | 42 | 10 | 0 | 22(1d/1d) | 10(1d/3d) |
| T6 | GXU2-1 | Het | 3d/2i | 42 | 11 | 0 | 21(2i/2i) | 10(3d/2i) |
| T7 | GXU1-1 | Het | 3d/2d | 42 | 10 | 0 | 22(3d/3d) | 10(3d/2d) |
| T8 | GXU1-1 | Het | 4d/3i | 42 | 11 | 0 | 22(3i/3i) | 9(4d/3i) |
| T1 | GXU30-1 | Bi | 1d/2i | 42 | 0 | 21(1d/2i) | 11(1d), 10(2i) | 0 |
| T2 | GXU35-1 | Chi | 1d/1i | 15 | 3 | 6(1d/2i) | 1(1d/1d) | 5(1i/2i) |
| T4 | GXU35-1 | Chi | 1i/5d | 15 | 7 | 1(1i/2d), 2(1i/2d) | 3(1d/2i) | 2(5d/5d) |

PT; number of plants tested, WT; wild type, Bi; bi-allelic, Homo; homozygous, Hetero; heterozygous, Chi; chimeric. d: deletion, i: insertion and WT: wild type. The numbers in front of the letters indicate the number of nucleotides affected. Corresponding mutations in two alleles are distinguished by '/'.

**Table S6.** Details about sequencing reads and quality output.

| Sample | Raw reads | Raw bases | Clean reads | Clean bases | Q20 (%) | Q30 (%) | NN (%) | GC content (%) |
|---|---|---|---|---|---|---|---|---|
| GXU7-1-1 | 10641415 | 6.38G | 10511127 | 6.30G | 98.28 | 94.60 | 0 | 47.68 |
| GXU7-1-2 | 10641415 | 6.38G | 10511127 | 6.30G | 97.42 | 92.22 | 0 | 48.44 |
| GXU7-1-3 | 10641415 | 6.38G | 10511127 | 6.30G | 98.26 | 95.23 | 0 | 48.22 |
| IR-96-1 | 10597095 | 6.35G | 10503372 | 6.30G | 98.33 | 94.69 | 0 | 46.92 |
| IR-96-2 | 10597095 | 6.35G | 10503372 | 6.30G | 97.60 | 92.58 | 0 | 47.47 |
| IR-96-3 | 10597095 | 6.35G | 10503372 | 6.30G | 96.56 | 95.63 | 0 | 46.33 |

Q20 and Q30: percentage of bases with a Phred value greater than 20 or 30, respectively, as a percentage of the total base; NN: percentage of unrecognized bases; GC content: calculate the sum of the number of bases G and C as a percentage of the total number of bases.

**Table S7.** Differentially expressed genes (DEGs) related to cytochrome P450 family, grain development and cell cycle.

| Locus/Gene Name | Annotation | log2 Fold Change |
|---|---|---|
| **Gene related to cytochrome P450 family** | | |
| *Os01g0377250* | Similar to Cytochrome P450 CYP71K15 | −1.78 |
| *Os01g0561600* | Cytochrome P450 family protein | −4.84 |
| *Os01g0628700* | Cytochrome P450 | −1.86 |
| *Os01g0858350* | Similar to cytochrome P450 | −1.91 |
| *Os03g0760300* | Cytochrome P450 family protein | −1.64 |
| *Os06g0639800* | Cytochrome P450 family protein | −8.08 |
| *Os10g0196000* | Similar to Cytochrome P450 CYP98A7 | −1.32 |
| *Os01g0858200* | Cytochrome P450 | 1.3 |
| **Genes related to grain size and development** | | |
| *Os01g0633100/GIF2* | Grain incomplete filling 2 | −1.43 |
| *Os02g0762600* | SG1-SHORT GRAIN1-related protein | −1.26 |
| *Os09g0517600* | Regulation of grain size | −1.95 |
| *Os03g0254400/OspPLAIIIα* | Patatin-related phospholipase A | −7.80 |
| **Genes responsible for cell cycle and division** | | |
| *Os02g0627800/ CycB1;4* | Rice cyclin gene | −3.93 |
| *Os04g0563700/CycB2;1* | Rice cyclin gene | 1.31 |
| *Os09g0466100/CycD4* | Rice cyclin gene | 6.28 |
| *Os08g0479300/CycD4;2* | Rice cyclin gene | 1.55 |
| *Os10g0563900/CycP4;1* | Cyclin-like gene | 3.23 |
| *Os01g0824700* | Cyclin-like F-box domain containing protein | −6.52 |
| *Os02g0105800* | Cyclin-like F-box domain containing protein | −2.00 |
| *Os02g0287900* | Cyclin-like F-box domain containing protein | 4.14 |
| *Os02g0535901* | Cyclin-like F-box domain containing protein | −1.22 |
| *Os01g0152950* | Cyclin-like F-box domain containing protein | −7.35 |
| *Os03g0708200* | Cyclin-like F-box domain containing protein | −1.58 |
| *Os04g0440300* | Cyclin-like F-box domain containing protein | −3.66 |
| *Os04g0479800* | Cyclin-like F-box domain containing protein | −2.32 |
| *Os05g0139200* | Cyclin-like F-box domain containing protein | −1.86 |
| *Os05g0575400* | Cyclin-like F-box domain containing protein | −2.56 |
| *Os07g0120400* | Cyclin-like F-box domain containing protein | −1.90 |

| | | |
|---|---|---|
| *Os08g0203800* | Cyclin-like F-box domain containing protein | −1.32 |
| *Os08g0461300* | Cyclin-like F-box domain containing protein | −1.66 |
| *Os09g0517800* | Cyclin-like F-box domain containing protein | −1.95 |
| *Os10g0124700* | Cyclin-like F-box domain containing protein | −1.38 |
| *Os10g0128600* | Cyclin-like F-box domain containing protein | −3.52 |
| *Os10g0155300* | Cyclin-like F-box domain containing protein | −1.43 |
| *Os11g0202300* | Cyclin-like F-box domain containing protein | −2.60 |
| *Os11g0208000* | Cyclin-like F-box domain containing protein | 1.45 |
| *Os11g0264200* | Cyclin-like F-box domain containing protein | 1.48 |
| *Os11g0584100* | Cyclin-like F-box domain containing protein | −1.40 |
| *Os05g0177100* | Cyclin-like F-box domain containing protein | −4.40 |
| *Os01g0380800* | Cyclin-like F-box domain containing protein | −1.56 |
| *Os11g0539700* | Cyclin-like F-box domain containing protein | −1.63 |
| *Os04g0544200* | Cyclin-related 2 domain containing protein | −2.73 |
| *Os08g0512600/CDKB* | Cyclin-Dependent Protein Kinase | 5.10 |
| *Os01g0367700* | Cyclin-dependent protein kinase-like protein | −1.2 |
| *Os03g0118400/cdc2Os-1* | Cyclin-dependent protein kinase | 3.16 |

**Table S8.** Differentially expressed proteins (DEPs) related to cytochrome P450, grain size and cell division.

| Protein ID | Locus/Gene Name | Annotation | Log2 Fold Change |
|---|---|---|---|
| **Proteins related to cytochrome P450 family** | | | |
| Q94IW5 | *Os01g0197100/CYP90D2* | Cytochrome P450 | 1.32 |
| Q6F4F5 | *Os04g0469800/D11* | Cytochrome P450 | −3.24 |
| **Proteins responsible for grain size and development** | | | |
| Q6YUX0 | *Os02g0747900/PGL2* | POSITIVE REGULATOR OF GRAIN LENGTH 2 | 1.45 |
| Q6ZIK5 | *Os02g0701300/GS2* | GRAIN SIZE ON CHROMOSOME 2 | 1.53 |
| Q6YYV8 | *Os08g0562500/ SLG* | BAHD acyltransferase-like protein gene | 1.62 |
| B9F4Q9 | *Os02g0244100/GW2* | E3 ubiquitin-protein ligase GW2 | −3.24 |
| Q6AT90 | *Os05g0139100/APG* | Transcription factor APG | −3.24 |
| **Proteins controlling cell cycle and division** | | | |
| Q0J4I1 | *Os08g0512600/CDKB2-1* | Cyclin-dependent kinase B2-1 | 1.79 |
| Q6YXH8 | *Os09g0466100/OscyCD* | Cyclin-D4-1 | 1.63 |
| P29618 | *Os03g0118400/cdc2Os-1* | Cyclin-dependent kinase A-1 | 1.55 |

**Table S9.** Primers used for RT-qPCR analysis.

| Gene name | Primer sequence (5'-3') |
|---|---|
| *Os03g0603100* | F: CTTCTCCGGAGCTTCAGGT<br>R: CTCCATTTCGAGGGACATGC |
| *Os03g0568400* | F: AGGGTGATGAAGAAGGCACA<br>R: GTCTCCCTGGGAACGAGTAG |
| *GL3.2* | F: AGTGGCATGTGAGAAGACCA<br>R: TTTCTCAGGCAGCAACAACC |
| *OsBADH2* | F: AGAGACGCTTGATTGTGGGA<br>R: ACAGTGGATAACTGGCCACA |
| *Os01g0737500* | F: ATCCTCCTTTGCACGATTGC<br>R: GATAGCTCCTGACGCAGTCT |
| *Os02g0439200* | F: TCCTCAACGCGGAAATCAAC<br>R: GGCAACCTTGGCTTTCTTGA |
| *Os03g0102300* | F: CTAACCTCGATTCCGTTCGC |

| | |
|---|---|
| | R: GAGATGAGCCTCCGGATGAA |
| *Os11g0293300* | F: GTCCTGTCTCGCTGTTTGTC<br>R: TCTTCCTGCTGGTGACAGAG |
| *Os12g0487500* | F: ACCCATGTGGTCGTACTTGT<br>R: ACGTACCGGGTGTACAGTTT |
| *Os03g0110400* | F: TTGTGCATCCCATGACCTCT<br>R: CTGTTGTTCCTTGGGTTGGG |
| *Os02g0465500* | F: AGATGGTGAAGGTGGTGGAG<br>R: GCAGGAATAAAGCAGCGGAA |
| *Os03g0281100* | F: GGAAGGCGAAGAAGCAGATG<br>R: TCACCCAGTAGAGCATGGTG |
| *Os10g0476400* | F: GCAATGCATGCCAACACTTC<br>R: CAAAGCTCCCATTGCTTTGC |
| *Os09g0287000* | F: GACGCCACAACGAAGATGAA<br>R: AGAAGGGAACATGGAAACGC |
| *PGL1* | F: CTCTCTCACCCTCTCTCCCT<br>R: CAGTGCCAACACAGAACCAA |
| *OsOS-9* | F: ATTGTTGCCGATGTCAGGTG<br>R: TTAAATGGCGCATCGACAGG |
| *GASR2* | F: GCACGAATACGATCCGTCTG<br>R: TAACAGCTCGTCAACACCCT |
| *Adh1* | F: GCGAATCCTTCCTTTCGCTT<br>R: AACTTCTCCACCTCCAGCTC |
| *OsCesA4* | F: CCCATGCCAGGACAATTAGC<br>R: ACCCTGTTCTTCCAGATGGG |
| *OsPAL5* | F: ACATCGGCAAGCTCATGTTC<br>R: CTTGAAGCCGTAGTCCAAGC |
| *CYP90D2* | F: ATGGGTGTGATGGTTAGGCA<br>R: GCGTTCCTGGGATTCAGTTC |
| *OsGLN2* | F: TGATTTCAGCCCACAGGCTA<br>R: TTTCACATGCGCCACTAACC |
| *OsTUB8* | F: CCTCTCTGGCTCTCTGCTAC<br>R: GATGACAACGAACGAAGGCA |

**Table S10.** Target sites with their positions, GC content and potential off-target score.

| Sr. No. | Target Sequence (5′-3′) | Position | Strand | GC % | Region | Off-target score |
|---|---|---|---|---|---|---|
| T1 | GATCTTCCGTGGCACAGACACGG | 3–22 | + | 55.0 | CDS | 0.234 |
| T2 | TTGACGACGCAGTGCAGGTACGG | 161–142 | - | 55.0 | CDS | 0.358 |
| T3 | GGGACCGGCTGCAGCACTCGGGG | 265–246 | - | 70.0 | CDS | 0.060 |
| T4 | CGATCGCCAGCGATCGCGCTGGG | 423–442 | + | 60.0 | CDS | 0.289 |
| T5 | CCATCTCCAGCGACATGTTCAGG | 421–440 | + | 75.0 | CDS | 0.381 |
| T6 | AGGCGCGCCCACGACAGGAGCGG | 750–769 | + | 75.0 | CDS | 1.000 |
| T7 | CAGCGGCAGCTCTTCGTCGCCGG | 15–184 | + | 70.0 | CDS | 0.242 |
| T8 | TACTTGGCCCGGACGGCGCCCGG | 475–456 | - | 75.0 | CDS | 0.288 |

T1-T1; represents the Target1 to Target8. Green highlighted are PAM regions. – and + represent the 5′-3′ and 3′-5′ direction of target sequence.

**Table S11.** Primers used for off-target analysis.

| Primer name | Primer sequence (5'-3') |
|---|---|
| POT1 | F: CCCTAAGTCAACCGGTCAAA |
| | R: GTGGCTAGGGAGTGGCAGT |
| POT2 | F: GATTTGGGATTTGGGGATTT |
| | R: GAATTTGCCGTCGATTCAGT |
| POT3 | F: TTCACCGACGTCCTTCTCTC |
| | R: TCCCTCGAAAGATCATCTCC |
| POT4 | F: TCTTTAGAGGGACGGACACG |
| | R: CTTTTGCTTGCTTGTTGCAG |
| POT5 | F: GACGACTCGCTCTTCAGCTC |
| | R: CGACCTGCTCAGTGTCTTGA |
| POT6 | F: AAGTCCGTCCCCTTGAAGTC |
| | R: CTACCTGCACCTCGTCATCA |
| POT7 | F: GCGTTGATGACGAGGTACAG |
| | R: GGGTTGAGGGATGGGGTA |
| POT8 | F: GAGTACACCGTCCCGGAGTT |
| | R: GTGCTCGACCTCCTCTCCT |
| POT9 | F: CTATGACCCCGATGCAAGAT |
| | R: CCATTGTGGAACCACATCAA |
| POT10 | F: TGAGGACGATGTGCAGGTAG |
| | R: ACCGCCTCTTCTCAAGTTCA |
| POT11 | F: TCCCTTCTGAAATTGCTGCT |
| | R: TGTGGGGGAAGACACTTAGG |
| POT12 | F: AAGGAAATGGACCGGAAGAT |
| | R: GCCTCCTCTCCTATCCCTCT |
| POT13 | F: GGTCAGGAACCAAAGGCATA |
| | R: GAGGATGTGAAGCAACGACA |
| POT14 | F: GTCATCCTCGTCAAGCCTTC |
| | R: GCCCCTACACATCAGCCTAT |
| POT15 | F: AGGAAAGGAATCACGACAGC |
| | R: CTCCTCCCTCCTCCTCCTC |
| POT16 | F: AGAGGTCAGAGGGGGCTAGA |
| | R: GATCGATCGCCCATTAGAAG |
| POT17 | F: AGGACTCAGGTCAGGTGCAT |
| | R: GAGAAGAAGGGGAGGAGGAA |
| POT18 | F: AACCACACCCACCATTTCTC |
| | R: GGTGGACAGGTACCAGAACG |
| POT19 | F: GCGAGCCTATGGTTCCTATG |
| | R: GAGAAACAAAGCAAAAAGGAAAA |
| POT20 | F: CACTCACCATTGTTGCCAAG |
| | R: GTCCCCAGGCTGTCGTAGT |
| POT21 | F: TTCACGCAGCTAACTGATGC |
| | R: TCGAGGTCGACTTCCTCTTC |
| POT22 | F: TTCACGCAGCTAACTGATGC |
| | R: GGAGACGTTCGGCCACTACT |

| | |
|---|---|
| POT23 | F: ATGTTCAGGGGGTTCCTGTA |
| | R: CAGTTCATGGTGTTGCCGTA |
| POT24 | F: CCGTTATGCTCACACACACC |
| | R: AAGGTTCTCCTGTCCCGTTT |
| POT25 | F: ACAGGCTCAGGCAAGAAGAA |
| | R: TCAACGCCTACAACGATCAG |
| POT26 | F: GATCGCTAGCTAGGCCGTTA |
| | R: GCTTGGCGAGAAGTAAGTGG |
| POT27 | F: ACAATGGCTGGACGACAAC |
| | R: CCAGCTAGCAGTCCAAGTCC |
| POT28 | F: ACCTCCCCGTAGATGAGGTC |
| | R: AGTACCTCCGCACCCTGGA |
| POT29 | F: TGCTGGAATCTGTGAATGGA |
| | R: GCCAAACAACGCTCCTGTAT |
| POT30 | F: GGGTACACCTCCGTGTCGT |
| | R: GCTGCTGCTCTCCTCGTC |
| POT31 | F: GGCGACCTCACCAACTTCTA |
| | R: CGTCACGAACGAATCAACAT |
| POT32 | F: CAGGAAAGTTCACGCAAACA |
| | R: CACTCATCGAAGGTGTGCAG |
| POT33 | F: CCTCCAACGATCCATCCAT |
| | R: TGCTGTTGTTGCTCCTGTTC |
| POT34 | F: AGCAGTCGTGGAAGAAGAGG |
| | R: CGACTCGTTTTTGCCTTCTC |
| POT35 | F: CCGTGGACGTTGAAGAAGAC |
| | R: CCCGTCACCTCCTACCTCTT |
| POT36 | F: TGGTCATGGCCCTTATGTCT |
| | R: ACAGCACTGTCAGCATGGTC |
| POT37 | F: AGCACAATCCTCGTCGATCT |
| | R: GCCATGCACGAGGAGATACT |
| POT38 | F: GACTCGCTTAATCGCTTTGG |
| | R: TGCGGCACATATATCGAGAG |
| POT39 | F: GATCGCCTTGAGGTACTTGG |
| | R: TTTGTGATCGGTGAAGGTGA |
| POT40 | F: CGGACGAACACACAAAGAGA |
| | R: ACGAGAGCTACAGCGAGGAC |

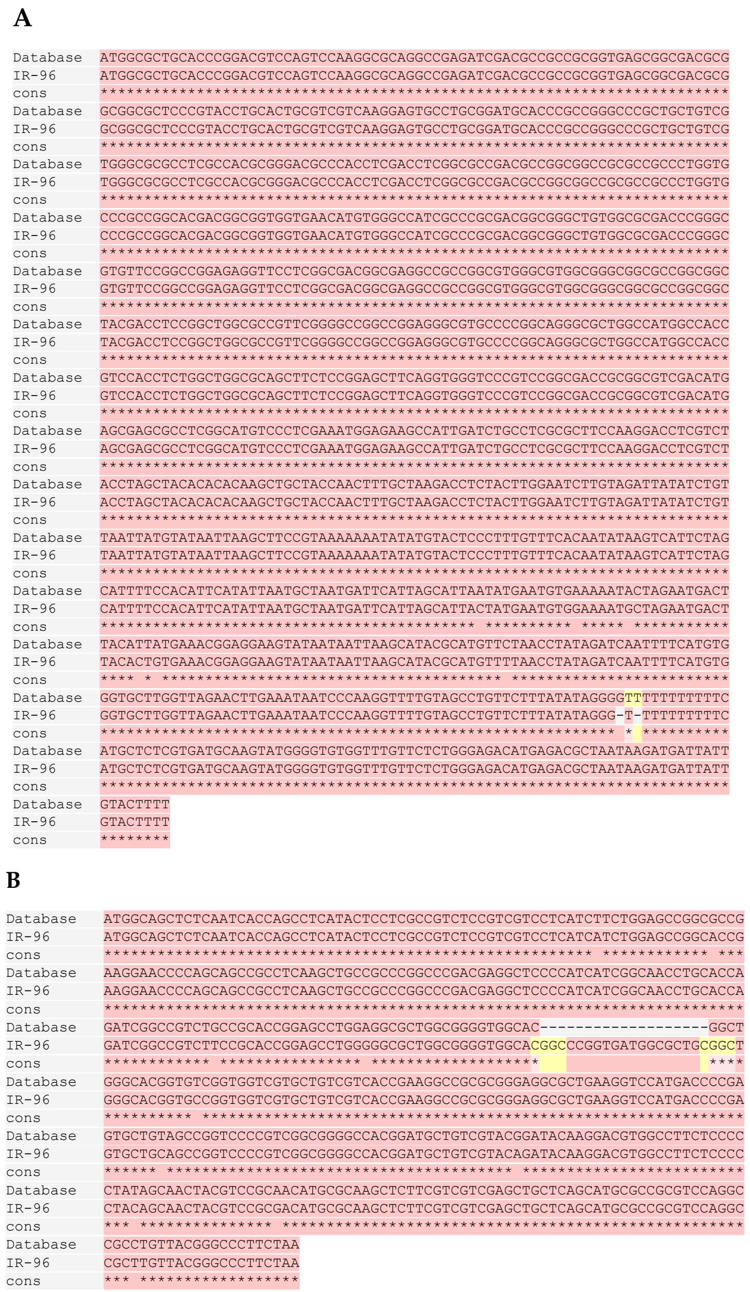


**Figure S1.** Sequence alignment of the (**A**) *Os03g0603100*, and (**B**) *Os03g0568400* gene in the reference genome and IR-96 rice variety. The SNPs between the reference genome and IR-96 are indicated with disappeared asterisks or dashes. The reference genome was used from Rice Genome Annotation Project website (http://rice.plantbiology.msu.edu/index.shtml).

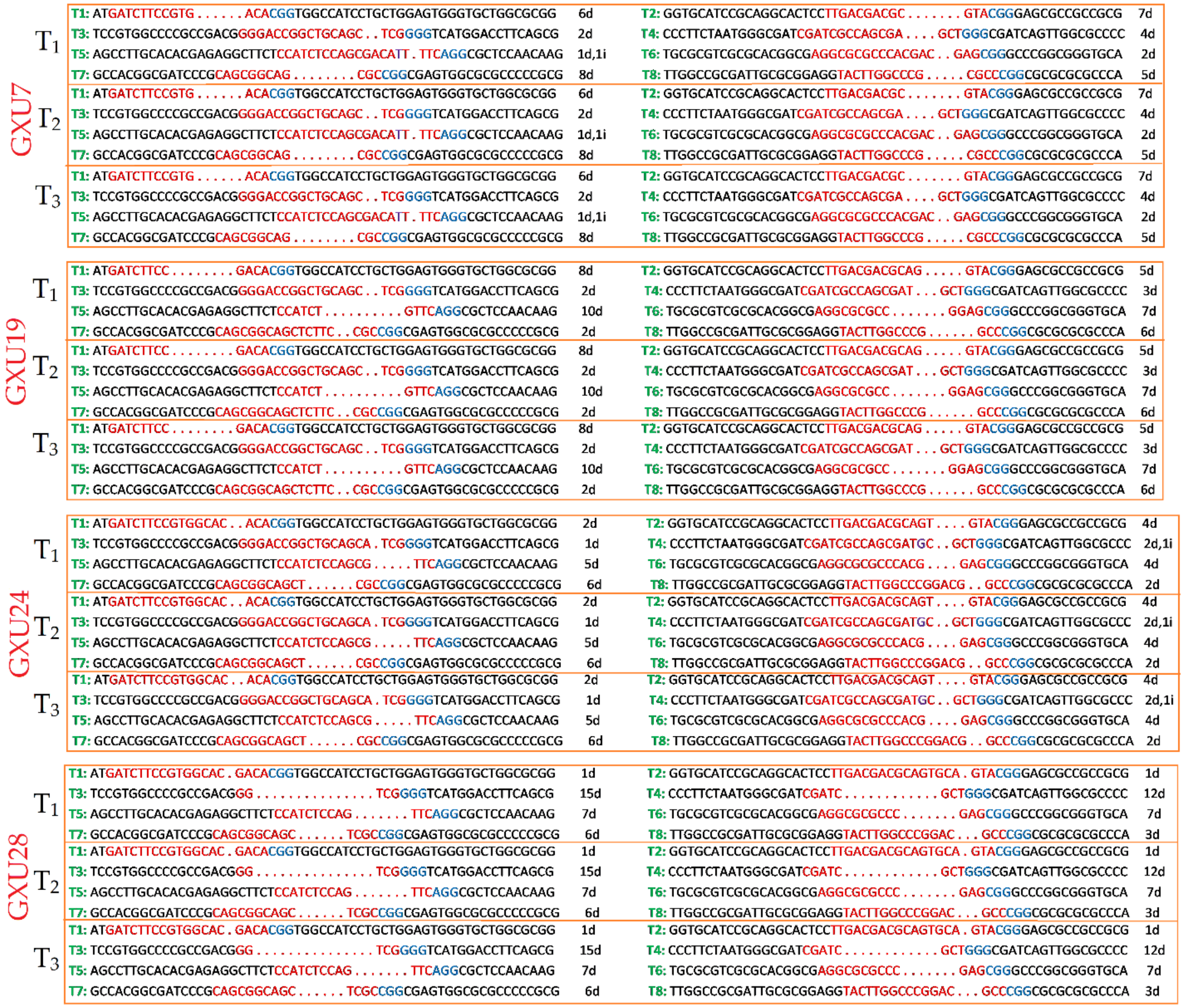


**Figure S2.** Transmission of mutations to subsequent generations in homozygous mutant plants.

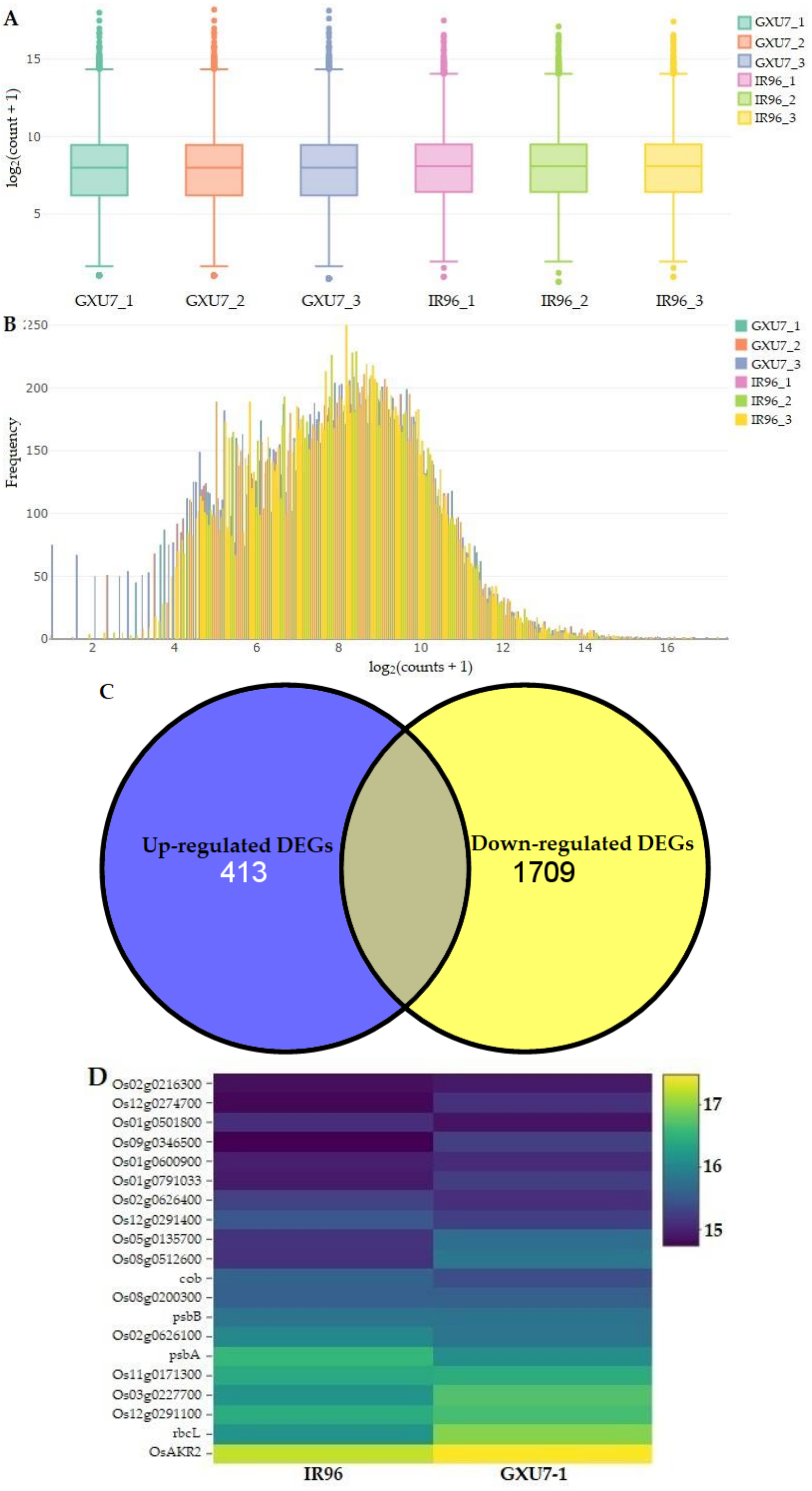


**Figure S3.** Transcriptome analysis of the wild type (WT) and mutant line (GXU7-1). (**A**) Box and whisker plot showing the plot for transformed read counts from transcriptome data of WT and GXU7-1; (**B**) Count data distribution frequency of transformed data from WT and mutant line; (**C**) Venn diagram showing the up-regulated and down-regulated differentially expressed genes (DEGs); (**D**) Heatmap of twenty most variable genes with higher mean expression values. Yellow color indicates gene IDs with a higher expression level and darker blue color represents a low expression level.

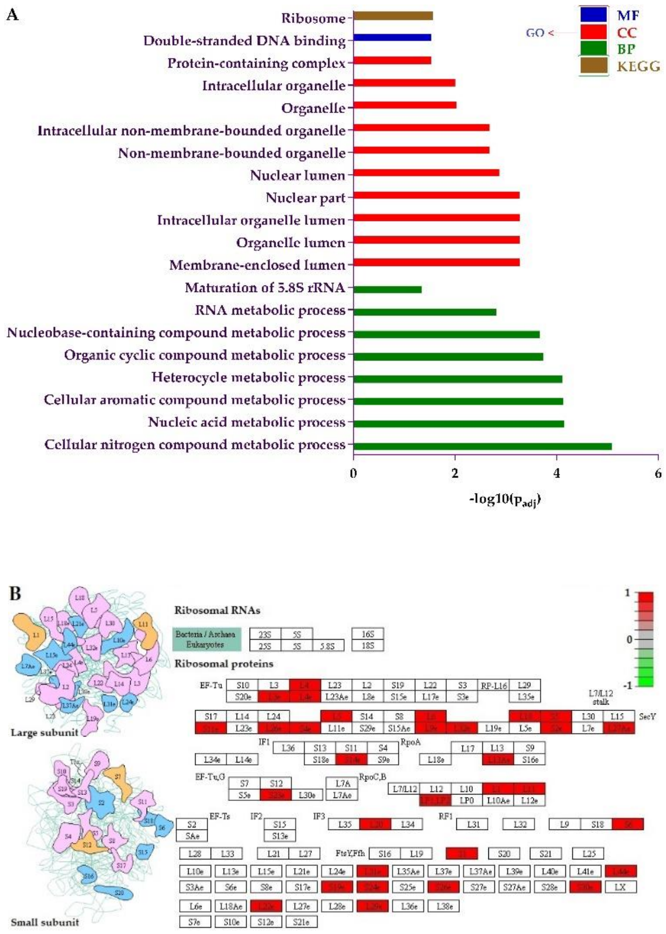


**Figure S4.** Gene Ontology (GO) and Kyoto Encyclopedia of Genes and Genomes (KEGG) pathway-analysis of differentially expressed genes. (**A**) Histogram showing GO terms and KEGG pathway, BP; biological processes, CC; cellular component, and MF; molecular functions; (**B**) A diagram showing ribosome pathway. Red highlighted genes were up-regulated, and those with white background indicate that the genes were not significantly up- or down-regulated in GXU7-1 compared with WT.

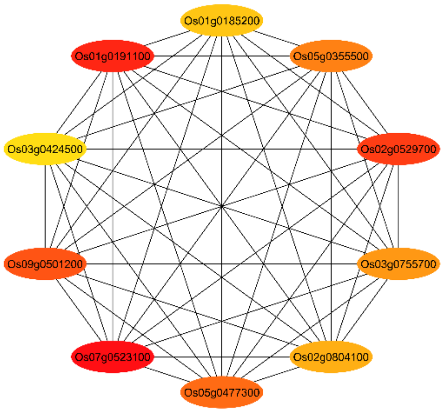


**Figure S5.** Top ten hub-genes selected from total differentially expressed genes (DEGs). Nodes represent genes, and edges indicate connections between the nodes. The redder color indicates a higher co-expression level.

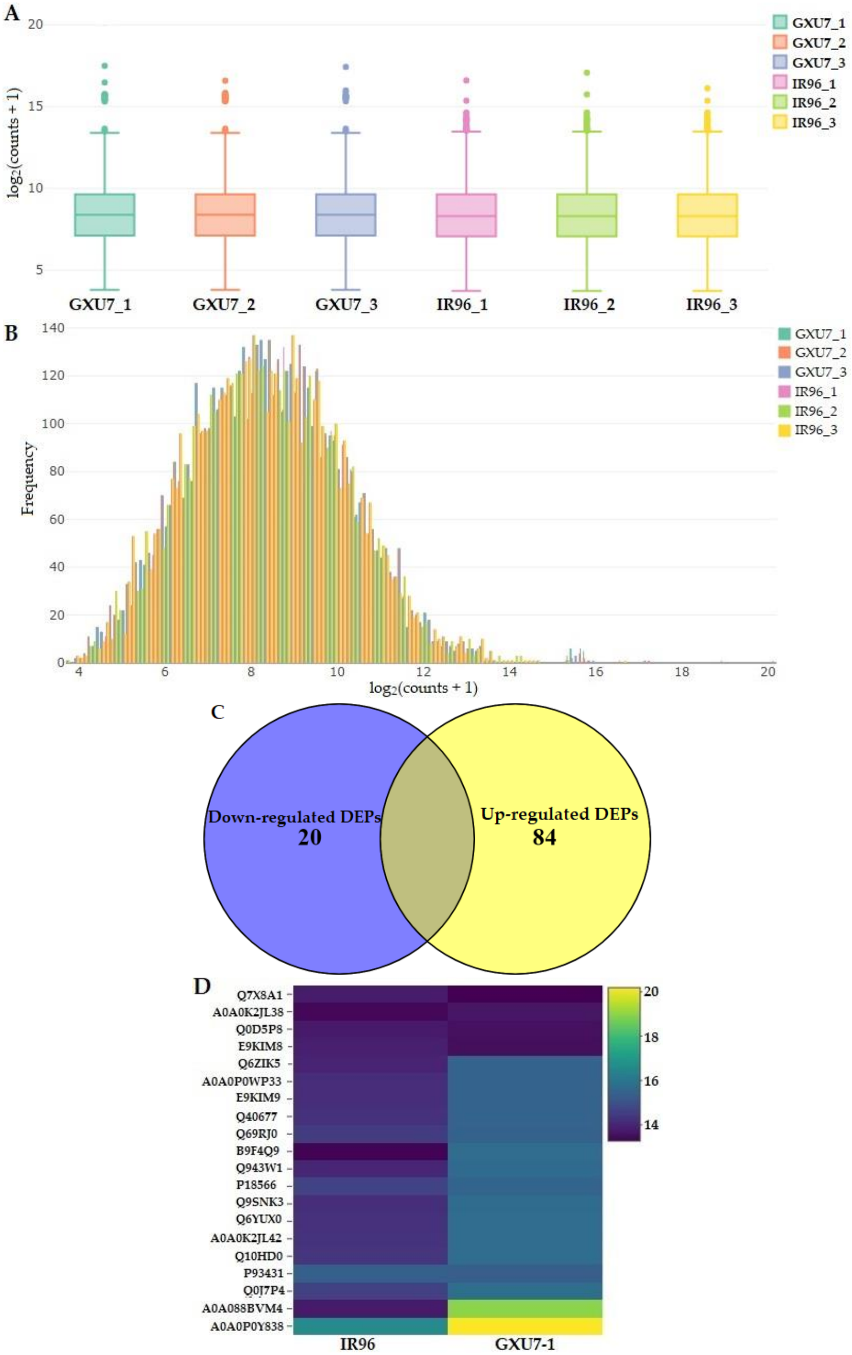


**Figure S6.** Basic information about proteomic analysis of the wild type (WT) and mutant line (GXU7-1). (**A**) Box-and-whisker plot of transcriptome count data distribution with $log_2$ values of the normalized abundance; (**B**) Histogram for count data distribution frequency of transformed data from WT and mutant line; (**C**) Venn diagram showing the up-regulated and down-regulated differentially expressed proteins (DEPs); (**D**) Heatmap showing twenty DEPs with highest mean expression level. Yellow color indicates a higher expression level and dark blue color indicates lower expression level.

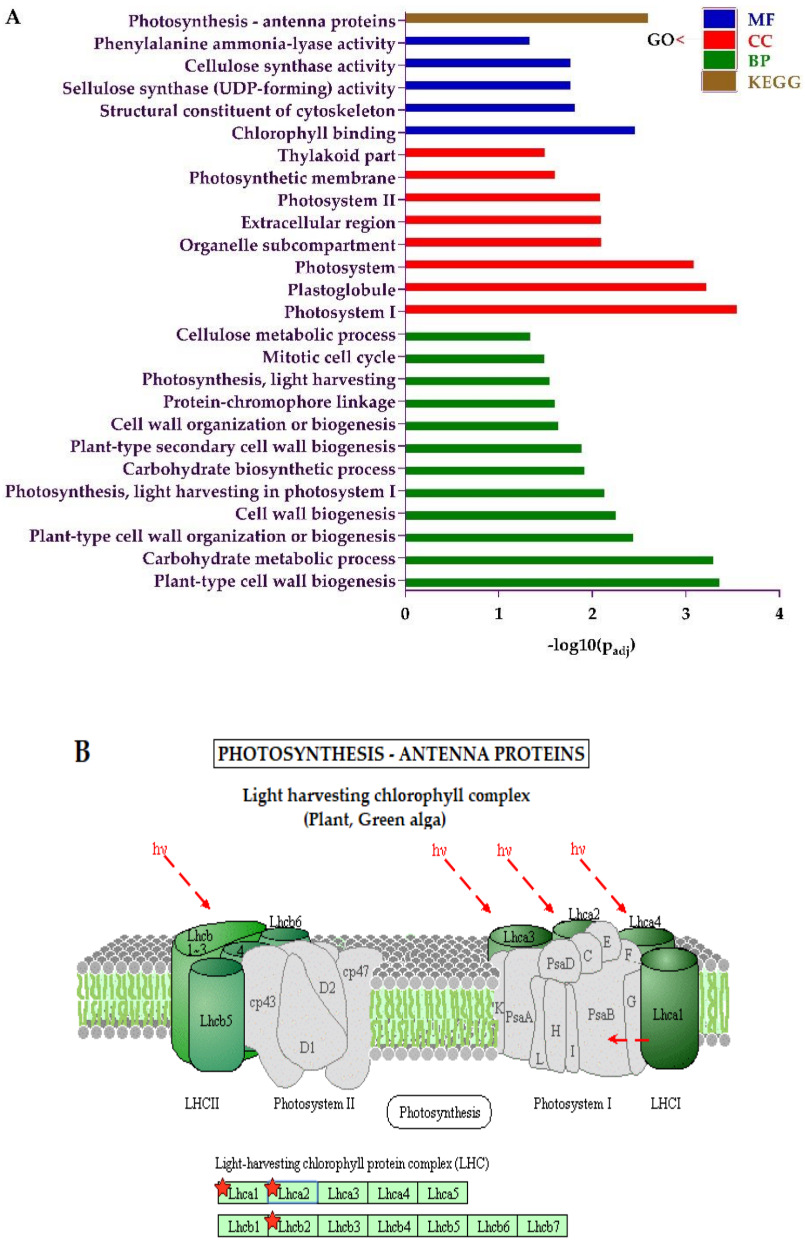


**Figure S7.** Ontology (GO) and Kyoto Encyclopedia of Genes and Genomes (KEGG) pathway-analysis, BP; biological processes, CC; cellular component, and MF; molecular functions; (**A**) Histogram showing GO terms and KEGG pathways; (**B**) A diagram representing light-harvesting chlorophyll complex pathway. Enzymes represented red stars indicate that the corresponding proteins were up-regulated, and other proteins were not significantly up- or down-regulated in GXU7-1 compared with WT.

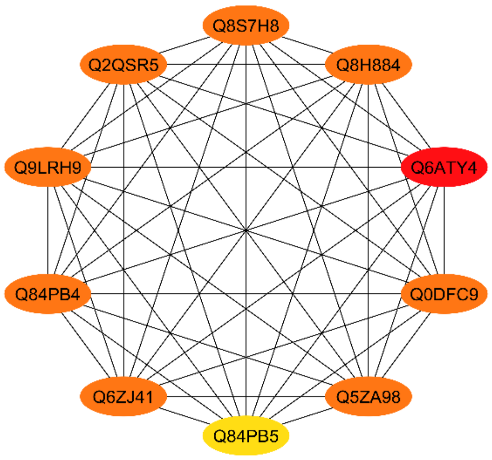


**Figure S8.** The top 10 hub-proteins positively correlated with wild type (WT) and CRISPR/Cas9 mutant (GXU7-1). The red color denotes the higher co-expression.

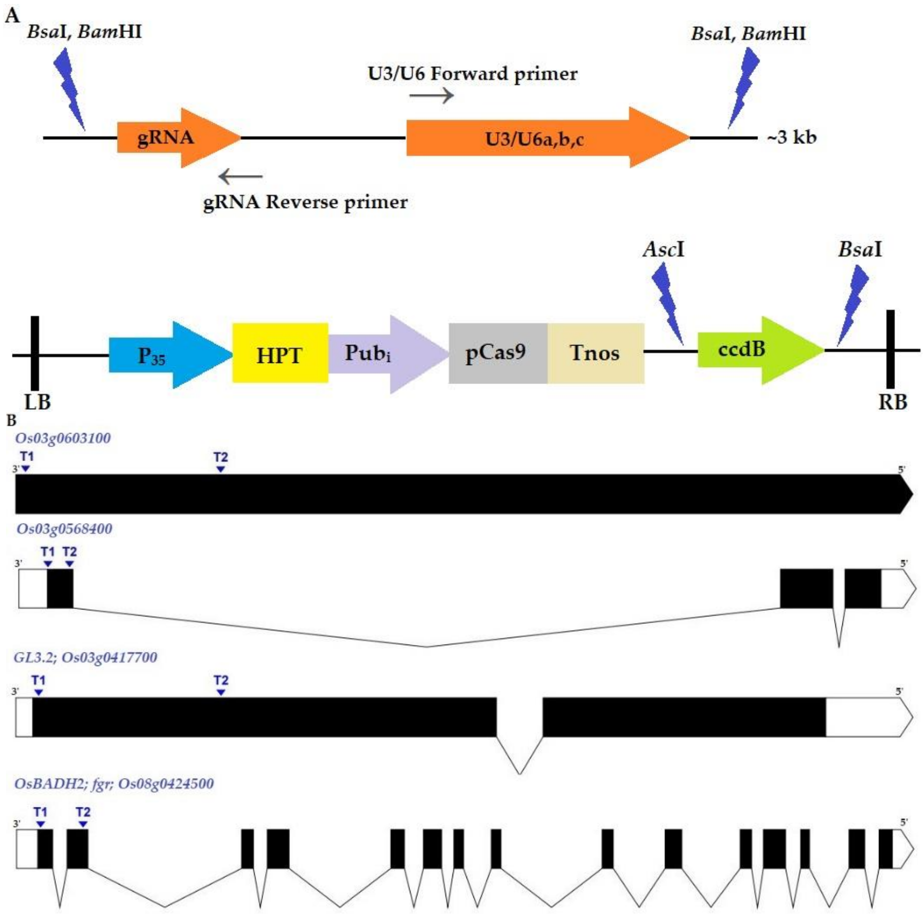


**Figure S9.** The map of vector and structure of four genes with positions of target sites. (**A**) Physical map of pYLCRISPR/Cas9Pubi-H vector. The CRISPR / Cas9 binary vector is approximately 16.5 kb in size and carries the resistance gene for hygromycin. It has left (LB) and right borders (RB), and from left to right, the *HPT* gene, the *Cas*9 driven by the ubiquitin promoter ($P_{ubi}$), the NOS terminator (Tnos), and the *ccdB E. coli* lethal gene. When assembling the target, restriction endonuclease *Asc* I or *Bsa* I can be used to produce distinct, non-palindromic sticky ends. Single guided RNA (sgRNA) is followed by the U3/U6 promoters for genome targeting; (**B**) Gene structure and gRNA positions of *Os03g0603100*, *Os03g0568400*, *GL3.2*, and *OsBADH2*. T1 and T2 represent Target1 and Target2, respectively.

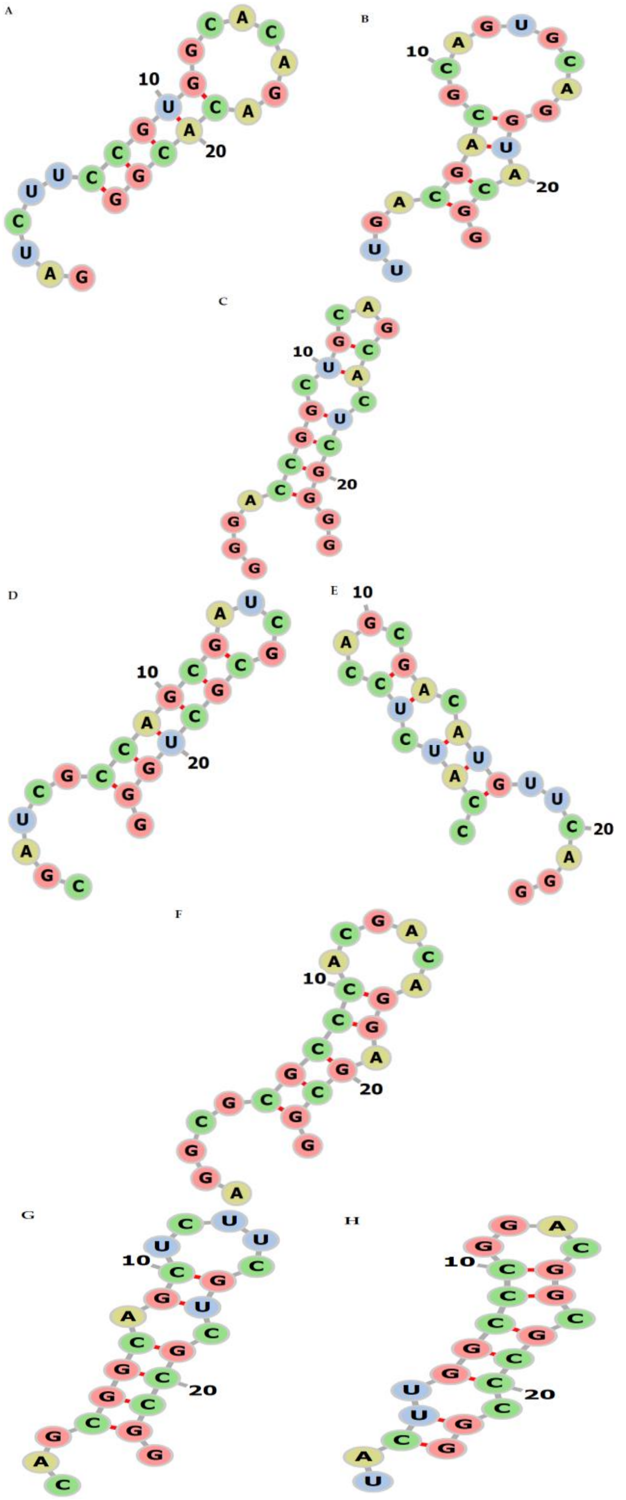


**Figure S10.** Schematic representation of secondary structures of (**A**) sgRNA1; (**B**) sgRNA2; (**C**) sgRNA3; (**D**) sgRNA4; (**E**) sgRNA5; (**F**) sgRNA6; (**G**) sgRNA7; and (**H**) sgRNA8 used in this experiment. The sgRNA structures shown above are colored by base-pairing probabilities. For unpaired regions, the color denotes the probability of being unpaired.
